# Toward 2D van der Waals Entropy Mixture MX_2_ (M = Mo, W; X = S, Se, Te) for Hydrogen Evolution Electrocatalysis

**DOI:** 10.1021/acsami.5c05482

**Published:** 2025-06-04

**Authors:** Jan Paštika, Deniz Güngen, Amutha Subramani, Vlastimil Mazánek, Marco Serra, Lunjie Zeng, Eva Olsson, Rui Gusmão, Zdeněk Sofer

**Affiliations:** † Department of Inorganic Chemistry, Faculty of Chemical Technology, 9306 University of Chemistry and Technology Prague, Technická 5, Prague 6, Prague 166 28, Czech Republic; ‡ Istituto Italiano di Tecnologia Via Morego 30, Genova 16163, Italy; § Department of Physics 11248 Chalmers University of Technology, Gothenburg SE-41296, Sweden

**Keywords:** Transition Metal Dichalcogenide, High Entropy Alloys, 2D material, Electrocatalysis, Hydrogen Evolution Reaction

## Abstract

High-entropy alloys have emerged as a class of materials, offering unique properties due to their irregular and randomized arrangement of multiple elements in an ordered lattice. This concept has been extended to two-dimensional (2D) van der Waals materials, including transition metal dichalcogenides (TMD), which exhibit promising applications in electrocatalysis. In this work, we have explored the synthesis of entropy mixture crystals (TMD_mix_) involving the chemical vapor transport of five individual elements, Mo and W as metal elements, S, Se, and Te as chalcogenide elements, resulting in a crystalline structure with a controlled composition Mo_0.56_W_0.44_(S_0.33_Se_0.35_Te_0.32_)_2_, with an estimated Δ*S*_mix_ of 0.96*R*. When observed along the [001] zone axis, STEM HAADF images indicate the presence of the different crystal phases of the 2D TMDs (1T, 2H, and 3R). Our findings demonstrate the potential of the entropy TMD_mix_ materials as catalysts for the hydrogen evolution reaction, offering an alternative to noble metal-based catalysts. To maximize the potential of TMD_mix_, we chose chemical exfoliation with the resulting material being subdivided into size groups, big and small, according to their lateral size. In an acidic medium, the lowest overpotential of 127 mV and a Tafel slope of 79 mV/dec were obtained for the exfoliated sample with a small lateral size (exf-TMD_small_).

## Graphical abstract

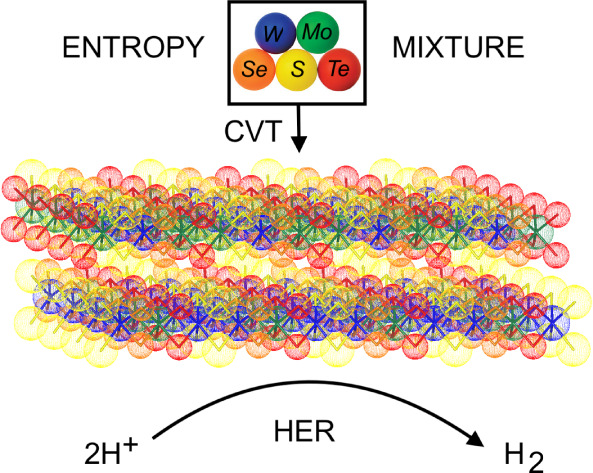

## 1 Introduction

High-entropy alloy (HEA) materials consisting of five or more elements, this class of material processing irregular and randomized arrangements of multiple atoms in an ordered lattice. HEAs were theoretically proposed in the 1980s and experimentally realized in 2004,^1^ with the report on single-phase solid-solution HEA nanoparticles, by thermally induced carbothermal shock (CTS) of precursor metal salt mixtures loaded onto carbon supports, paving the way for further exploration and applications of such materials.^2^ The design concept strategy for HEA has also been adapted to 2D van der Waals materials (HE2D), leading to the discovery of dichalcogenides,^3,4^ phosphorus trichalcogenides,^5,6^ halides,^7^ oxides,^8^ layered double hydroxides,^9^ and MXenes.^10^ From a thermodynamic point of view, the formation of HE2D is the result of competition between enthalpy and entropy (Δ*G*_mix_ = Δ*H*_mix_ – *T*Δ*S*_mix_).^11^ The increase in Δ*S*_mix_ lowers Δ*G*_mix_. This promotes the formation of a stable single-phase solid-solution structure. In accordance with the Boltzmann hypothesis, the Δ*S*_mix_ in 2D materials with cationic and anionic sites is calculated by considering the contributions from both sublattices.^12^ For a material to qualify as HEA, Δ*S*_mix_ must exceed 1.61*R*.^13^ Additionally, the material must contain at least five elements, and atomic percentages of these elements range from 5% to 35%.^12^

For electrocatalysis, the effect of the lattice distortion effect improves the coordination environment of the atoms on the catalyst surface or the adsorption energy of the intermediates.^14^ Beyond the usual advantages introduced by 2D materials, their characteristic disordered structure opens up a wide range of variability in the local atomic arrangement which allows to introduce a complex array of diverse catalytic sites on a single material.^15^ An example of this effect is shown by the system TiVZrNbHf, which is able to absorb a higher amount of hydrogen molecules compared to its constituents, thanks to the absorption taking place in both tetrahedral and octahedral sites introduced by the increased lattice strain.^16^ The HEA materials are characterized by a complex lattice distortion pattern typical of nonequilibrium states, with their surface considered as a composite work-function mosaic along the crystalline planes, leading to a shift in the d-band center.^15^ Therefore, when an atom is coordinated into a vacancy, its^17^ diffusion to adjacent tiles results in being hindered by a local energetic barrier acting as a trap for the coordinated species, thus, enhancing their interactions.^18^ These features have been further exploited for electrochemical energy conversion applications such as water-splitting reactions,^3,19–22^ methanol oxidation,^23^ nitrogen reduction reaction (NRR),^19^ and carbon dioxide reduction (CO_2_RR).^24^

Challenges such as complex optimization of composition, phase stability, and catalytic activity still must be further developed.^25^ Multiple principal elements in HE2D can provide a variety of catalytic sites with different electronic and chemical properties, and lattice distortion caused by the different sizes of atoms that make up HE2D facilitates the transportation of active species.^26^ The interplay of features such as severe lattice distortion, sluggish diffusion, cocktail effect, and high entropy can result in the appearance of synergistic properties.^27^ The application of HE2D properties to catalysts can lead to longer lifetimes and better performance under harsh reaction conditions, such as high temperature and high current density.^28^

The work presented herein is focused on the synthesis, exfoliation, and electrochemical application as a catalyst for HER of entropy mixture MX_2_, where M represents the metal elements Mo and W, and X represents the chalcogen elements S, Se, and Te. The starting synthesized material, TMD_mix_ was exfoliated and divided into exf-TMD_big_ and exf-TMD_small_ samples according to the flakes’ lateral size. These TMD samples were investigated for their electrochemical performance and structural composition. The exf-TMD_small_ showed promising performance together with lower overpotential, and subsequent measurements of electrochemical stability demonstrated the application of the material as a catalyst for the electrochemical cathodic water-splitting reaction.

## 2 Results and Discussion

### 2.1 Characterizations

The synthesis of entropy transition metal dichalcogenide mixture (TMD_mix_) MX_2_ bulk crystals was performed by the chemical vapor transport (CVT) of the five individual elements (more details in the Experimental Section and Methods). This approach has been previously proven effective for the growth of high-quality 2D layered MX_2_ crystals,^29^ ranging from single to quaternary metal sulfides, or TMD constituted of binary mixtures of chalcogen elements.^30^ In this case, the procedure was adapted to attempt the preparation of a TMD_mix_ constituted simultaneously by a binary mixture of d-metal elements and ternary chalcogenides. The powders of the five individual precursors were combined in equal ratios for cation and anion, and after the formation of bulk powder, chemical vapor transport crystal growth was performed, which yielded wide crystals.

The morphology of TMD_mix_ was observed by an optical microscope (Figure S1a) and scanning electron microscopy (Figure 1a). As shown in the SEM micrograph, the size of obtained TMD_mix_ crystals has a plate-like morphology that varies in a range of tens up to several hundreds of micrometers. The crystals consist of a large number of multilayered, well-defined agglomerated flakes with occasional smaller particles distributed on their surfaces, as exemplified in Figures 1b and in the overlay of the SEM-EDX elemental mapping in Figure S1b. The representative EDX spectrum is shown in Figure 1c, which confirms the presence of the expected elements with a 1.39 keV signal corresponding to Se Lα and 1.78 keV corresponding to W Mα. The broad peak centred at 2.30 keV represents the overlapping Mo Lα and S Kα transitions. The higher energy spectral range has emission lines due to Te Lα and Te Lβ transitions.

**Figure 1:**
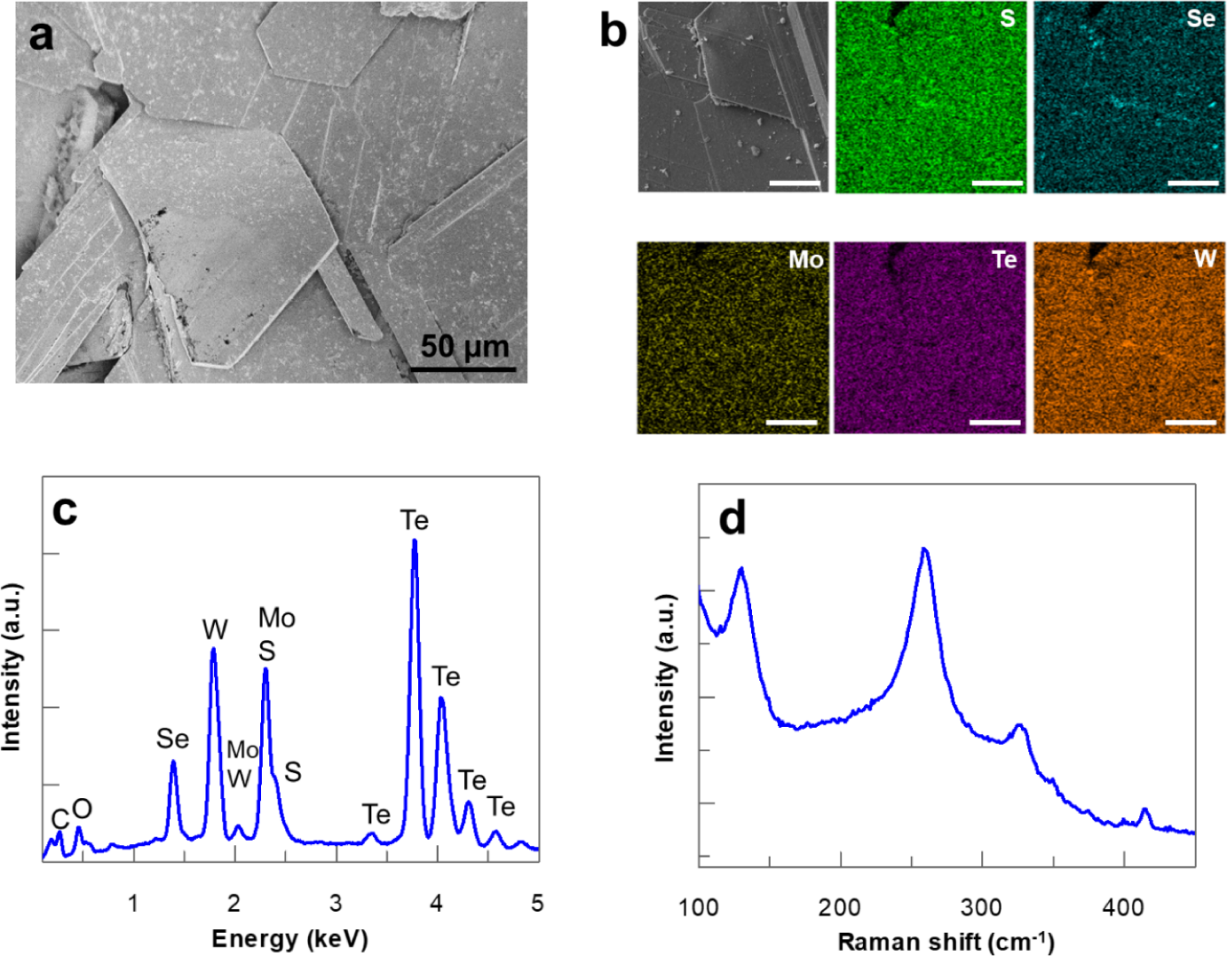
Characterization of synthesized TMD_mix_ crystals. a) Combined in-beam SEM and in-beam BSE modes micrograph. Scale bar represents 50 μm. b) SEM micrograph and corresponding EDX elemental distribution maps of constituent elements: S (green), Se (blue), Mo (yellow), Te (magenta), and W (orange). Scale bar: 25 μm. c) Example of acquired EDX spectra. More EDX acquisition is shown in the SI. d) Raman spectrum using 532 nm LL.

The EDX spectroscopic analysis, conducted across multiple localized spots (30–300 μm view field), revealed consistent detection of all expected elements, with Te predominating in the stoichiometric distribution. Figure S2 shows the spectra of distinct regions, demonstrating statistically some extension of compositional heterogeneity along the multiple crystal plates. Further corroboration is provided in Figure S3, which displays additional wide-field domains with the corresponding Point & ID EDX spectra. The statistical at. % of the compositional variance of the synthesized material obtained from the EDX average quantification of all the different random flakes and crystal plates shown across different regions (Table S1).

For a more accurate determination of composition, the elements were quantified using inductively coupled plasma optical emission spectrometry (ICP-OES), as presented in Table S2. From the wt % quantification, molar quantities were obtained from the atomic mass normalization, which point to a molar ratio Mo/W 1.2, a M/X ratio of 0.48, which is close to the expected value of 0.5, and for the ratio between chalcogenides of S:Se:Te = 0.97:1.0:0.89. From the ICP-OES results, the values of the chemical stoichiometry of the TMD_mix_ were estimated as Mo_0.56_W_0.44_(S_0.33_Se_0.35_Te_0.32_)_2_. Each of the five elements was quantified according to the empirical criterion of a minimum of 5% and based on the equation shown in SI, the estimated Δ*S*_mix_ was estimated at 0.96*R*. This Δ*S*_mix_ value is below the lower limit for HE mixtures; however, TMD_mix_ can be described as a medium-entropy mixture material (0.69*R* < Δ*S*_mix_ < 1.61*R*).^11,12^ Additionally, the parameters of bond length variance (β), atomic size mismatch (Δδ), and Pauling electronegativity deviation (Δχ) were calculated for TMD_mix_, for which the equations are shown in SI. The Δχ of 16% complies with the critical Δχ lower than 15–20% criterion for solid solution stability.^11^ This electronegativity compatibility minimizes charge transfer instabilities and reduces the sublattice segregation driving forces. The Δδ value of 11.7% also complies with the Hume–Rothery stability criteria for the atomic radius ratio factor of less than 15%,^31^ which maintains random substitutional disorder and prevents thermodynamically driven phase separation in discrete intermetallic assemblies. The obtained value of 5.1% for β, point to partial phase segregation susceptibility while exceeding the Hume–Rothery thresholds of less than 2%, remains within the stabilization range for other reported HE2D.^32^ This moderate distortion may generate beneficial lattice strain effects that can enhance the catalytic activity through electronic structure modulation.

The Raman spectrum of the synthesized TMD_mix_ (Figure 1c) has four distinctive peaks corresponding to the in-plane (E_2g_) and out-of-plane (A_1g_) phonon modes of layered TMDs.^33^ Based on the literature,^29,34^ the peak at 132 cm^– 1^ is associated with Td-WTe_2_ Raman spectrum, identified as tilted out-of-plane A_1g_ modes. The peak at 256 cm^– 1^ is related to the E_2g_ resonances of the M-Se bonds, while the peak at 327 cm^– 1^ is attributed to in-plane E_2g_ resonances of the W–S bonds. Finally, the peak centered at 414 cm^– 1^ relates to the out-of-plane A_1g_ phonon modes of M–S bonds. Some observed shifts in the Raman spectrum compared to literature values for known materials can be attributed to the fact that the synthesized TMD_mix_ is not a single MX_2_ material but a combination of the five elements. While TMD_mix_ shares some characteristics with the known materials MX_2_, the specific atomic arrangement and bonding cause slight variations in the vibrational frequencies and peak positions and overlaps in the Raman spectrum. These variations are consistent with the Raman-active modes of the *D*6_*h*_ point group and *P*6_3_/*mmc* space group that define the 2H-phase TMD structure.^34^ The additional A_1_g mode of Td-WTe_2_, indicative of orthorhombic Td (1T’) phase 132 cm^– 1^ mode, reflects contributions from Td-phase domains, indicating some level of structural heterogeneity at the nanoscale.^35^

The crystal structure of TMD_mix_ was analyzed by X-ray powder diffraction (XRD), as shown in the pattern in Figure 2, along with the comparison of the MX_2_ powder diffraction file (PDF). The diffraction peaks are indexed to the individual MX_2_, with a predominance of the 2H phase, while matches with 1T and 3R phases were also verified. Splitting of (002) reflection indicates local deviation in composition, which corresponds to slight changes in the interlayer distance. This also correlates with the local changes in composition observed by EDS (Figure S1e). It should be noted that when comparing the entropy material with individual MX_2_ patterns, several elements of different sizes occur in the lattice positions and inducing a lattice distortion effect characteristic that causes shifts upon the 2θ reflections.^30^ Major diffraction peaks are registered between 12.5° < 2θ < 14.0°, related to the (002) lattice planes of rhombohedral MoSe_2_ (ICDD 04-005-6605), monoclinic MoTe_2_ (ICDD 04-007-1140), orthorhombic WTe_2_ (ICDD 00-024-1352), and hexagonal crystal systems for MoS_2_ (ICDD 04-002-9908), WS_2_ (ICDD 04-002-9909) and WSe_2_ (ICDD 04-001-9282). The estimated average size of crystallites is 46.4 nm, as calculated using the Scherrer equation.^36^ This value points out how each phase can be homogeneously dispersed in the entropic mixture, which corresponds with the EDX measurement. The obtained crystallinity index (CI)^37^ of TMD_mix_ was 95.8%, demonstrating that the material has a predominantly ordered crystalline microstructure.

**Figure 2:**
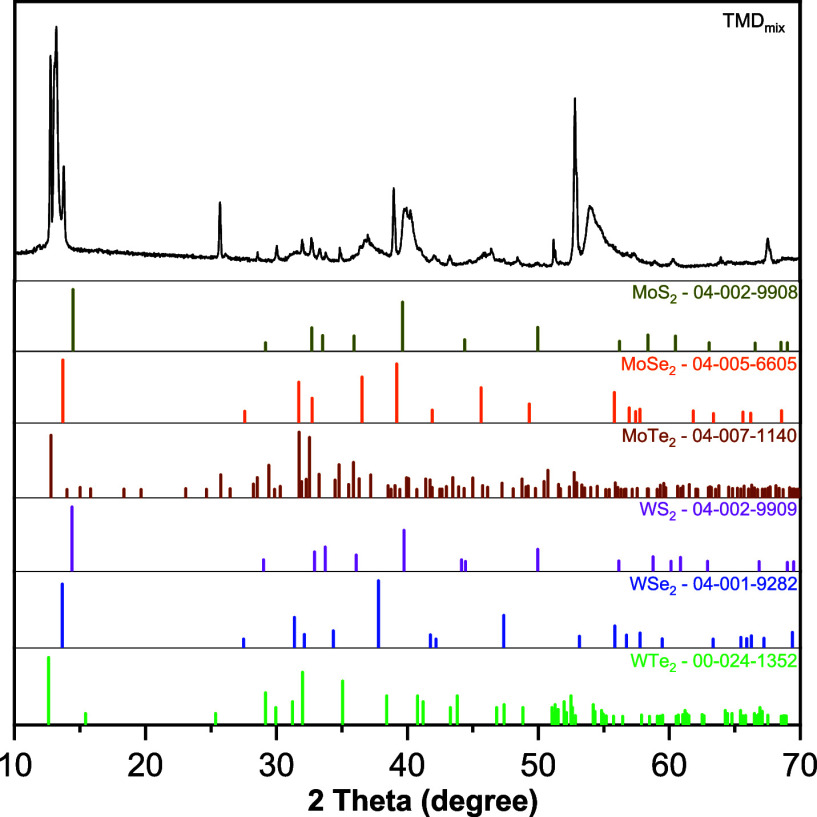
Powder XRD pattern of TMD_mix_ sample compared with PDF reference patterns for MX_2_ TMDs phases, with M = Mo, W and X = S, Se, Te.

STEM-EDX mapping of elements confirms that the material consist of Mo, W, S, Se, and Te (Figures 3a–3f and S1c). The spatial microdistribution of the TMD_mix_ constituent elements tests was investigated for a wider flake. The five elements are distributed over the whole flake, although there is some level of nanoscale inhomogeneity in their distributions, as observed in the line scan analysis of the flake (Figure S1d and e). Atomic resolution STEM HAADF imaging clearly shows the atomic structure of the flake viewed along the out-of-plane direction (the [001] zone-axis). The 2D sheets have a single-crystalline structure, evidenced by the uniform atomic arrangement pattern shown in the STEM images (Figure 3f). When viewed along the [001] zone-axis, the STEM HAADF images of the different phases of TMDs (1T, 2H, and 3R) show different patterns, which can be used to identify the crystal phases of the 2D TMDs. The single-crystalline 2D flakes used in this study are in the 3R phase, as revealed by comparing the experimental images with the simulated results (Figure S4). STEM HAADF image intensity scales with the atomic number of the material under electron beam illumination, roughly by *Z* ^2^. The variation in the image intensities of the atomic columns is thus due to the distribution of the transition metal and chalcogenide atoms. For example, the brightest areas in the image likely indicate that there are more W atoms in those atomic columns (Figure 3f). Apart from the 3R structure, the atomic-scale image intensity variation does not possess extra well-defined periodicity, suggesting a random distribution of the elements in the lattice. The observed crystallographic heterogeneities represent localized structural variations rather than compositional segregation, indicating that size-mismatch effects, also derived from β and δ estimation, manifest as crystallographic polymorphism (2H, 1T, and 3R) while maintaining chemical homogeneity. Further examples of STEM-EDX are shown in Figure S5, which provides a microscale compositional analysis of randomly selected sheets. The observed elemental distribution mapping points to compositional variations within discrete domains, while there does not seem to be preferential chalcogenide partitioning or elemental segregation between adjacent sheets.

**Figure 3:**
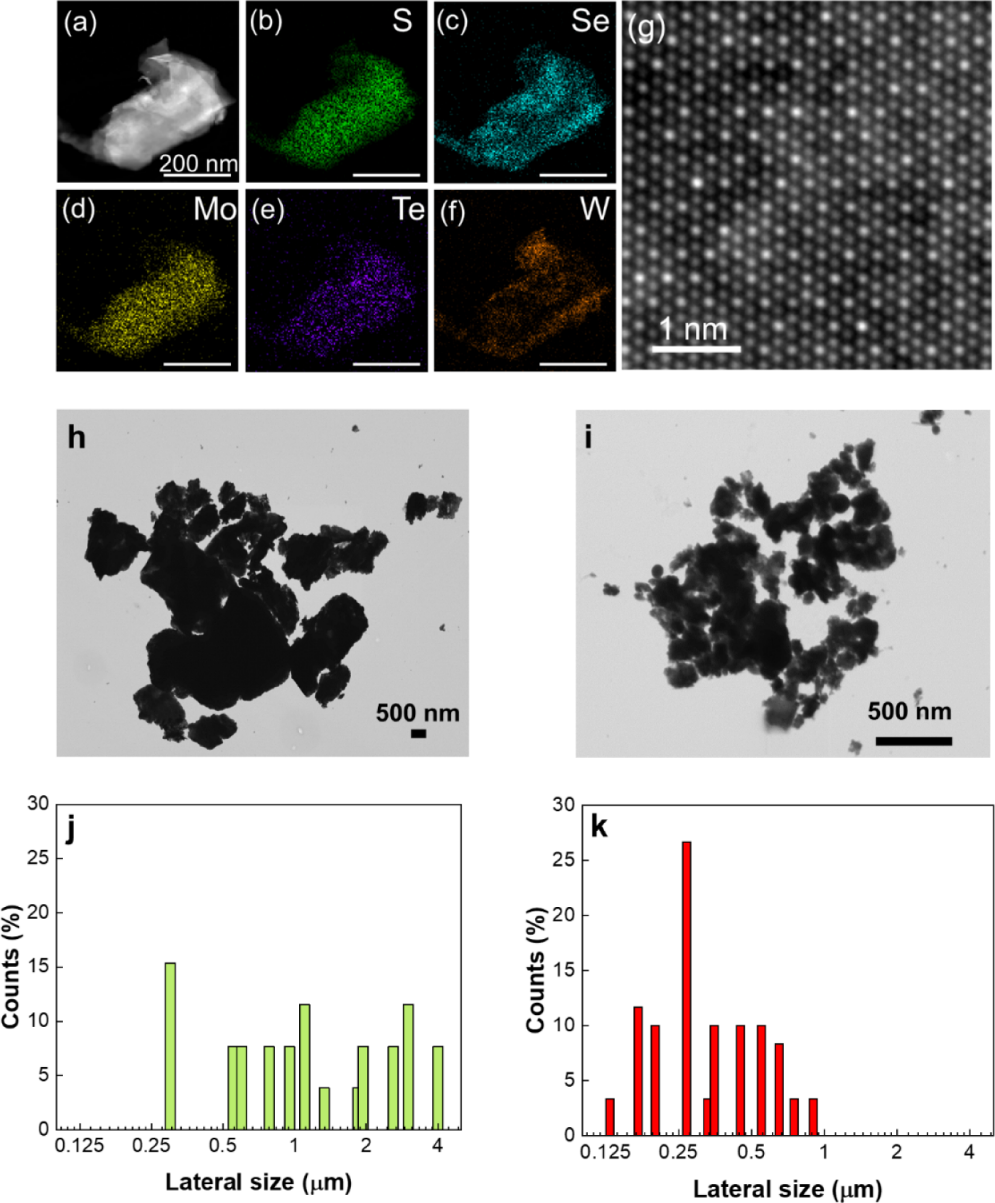
Elemental distribution and atomic structure of the 2D TMD_mix_ sample revealed by STEM-EDX elemental mapping and STEM HAADF imaging. (a) STEM HAADF image of a single crytalline flake. (b–f) Simultaneously acquired elemental maps of S, Se, Mo, Te, and W, respectively. (g) An atomic resolution STEM HAADF image of the flake, showing the 3R structure pattern. Bright mode STEM images of (h) exf-TMD_big_ and (i) exf-TMD_small_. Histograms showing lateral size statistics measured from STEM micrographs for (j) exf-TMD_big_ and (k) exf-TMD_small_.

The chemical exfoliation of TMD_mix_ crystals was carried out with *n*-butyllithium, with top (exf-TMD_small_) and bottom phases (exf-TMD_big_), being separate after the centrifugation cycle. The separate phases were observed by STEM (Figure 3h,i) in order to measure the sheets lateral size distribution (Figure 3j,k) and to control the presence of elements by EDX mapping (Figure S6a and b). STEM measurements point to exf-TMD_small_ having an average lateral size of 0.43 μm and exf-TMD_big_ 1.58 μm.

The surface of exf-TMD_small_ was also characterized by X-ray photoelectron spectroscopy (XPS). Core-level detail spectra were deconvoluted to individual bonding state components. Each element is composed of a single bonding state corresponding to the metal dichalcogenide. In the range between 60 and 25 eV, as shown in Figure 4a, there is an overlay at lower binding energies of Te 4d, Mo 4p, and W 4f core levels, and the peak at higher binding energies is related to Se 3d. Only molybdenum showed a second bonding state at higher binding energies that can correspond either to oxide (MoO_2_) or trichalcogenide (MoCh_3_).^38^ For the range between 222 and 240 eV, the ratio of Mo^IV^ 3d (A) to Mo^IV^ 3d (B) bonding states is 5:1 (Figure 4b). It is assumed that molybdenum is more likely in the form of trichalcogenide since there was quite a small amount of oxygen (27 at. %) which can mainly originate from adsorbed species together with carbon (31 at. %), from adsorbates like H_2_O, CO_2_, or organic molecules. Moreover, selenium and tellurium are present as chalcogenides although both of them are far more susceptible to oxidation compared to other elements. The regions of the spectrum corresponding to S 2p and Te 3d with the respective deconvolution and assignment of components are shown in Figure S7.

**Figure 4:**
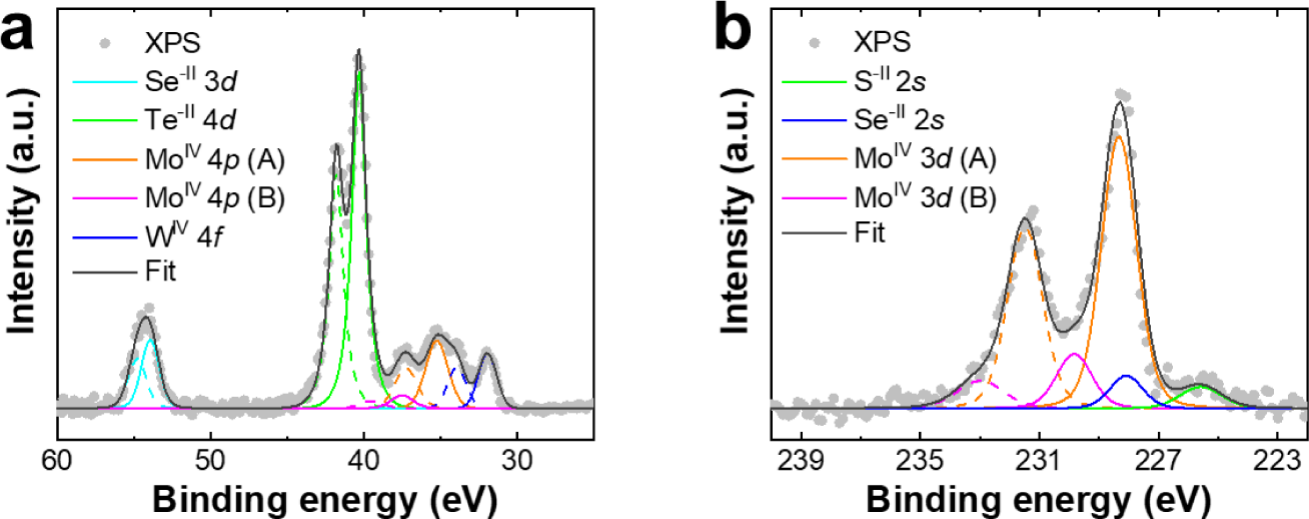
High-resolution XPS spectrum of exf-TMD_small_ ranging from (a) 60–25 eV and (b) 240–222 eV.

### 2.2 Hydrogen Evolution Reaction

The electrochemical performance of TMD materials toward the hydrogen evolution reaction (HER) in an acidic medium (0.5 M H_2_SO_4_) was initially screened by linear sweep voltammetry (LSV). The commonly used direct indicators of the material’s intrinsic activity are the overpotential at a current density of −10 mA cm^–2^ (η_10_), corresponding to a value close to 10% solar-to-chemical efficiency of HER, and the Tafel slope (TS), which correlates to how much overpotential is necessary to achieve a certain current density. Figure 5a shows the LSV curves of the materials and the benchmark catalyst 10% Pt/C. It can be inferred that the TMD_mix_ exhibits relatively high HER η_10_ at 289 mV. On the other hand, exfoliated phases register a significant improvement compared to the starting TMD_mix_. The exf-TMD_big_ can provide superior catalytic activity with a η_10_ of 150 mV, surpassed by the value obtained for exf-TMD_small_, having a very promising η_10_ of 127 mV, which is the closest to the value registered for 10% Pt/C (82 mV). From the extrapolation of the linear region of the overpotential vs log *j* (Figure 5b), the TS measured for one full decade was obtained to understand the electrochemical kinetics of these materials. The TS for exf-TMD_small_, with a value of 79 mV/dec, was also the closest to the TS of 10% Pt/C (35 mV/dec); thus, this was selected as a catalyst for HER for further studies. The determined TS for exf-TMD_small_, suggest that the rate-determining step (RDS) corresponds to the Volmer–Heyrovský mechanism (40 < TS < 120 mV/dec), involving proton adsorption step and electrochemical desorption. In contrast for the TMD_mix_ with a TS of 137 mV/dec, the RDS suggests corresponds to the Volmer step. The mapping of the TS and η_10_ values (Figure 5c) confirms that exf-TMD_small_ performs better for HER in an acidic medium among the synthesized materials, and even when comparing its performance when the current is normalized with the performance among other TMD and HE2D materials reported in the literature. The concept of mass activity (MA), when current is normalized with the material, is used as a complementary marker to the areal activity. The values obtained for the MA at −100 mV vs RHE follow the same trend as η_10_ with exf-TMD_small_ having the highest value at 6.9 A/g, followed by exf-TMD_big_ at 3.4 A/g, and TMD_mix_ with the lowest value of 1.3 A/g. When the current density is 1 mA/cm^2^, the first term in the Tafel equation that defines the TS becomes 0, from which the exchange current density (*j*_0_) can be estimated, pointing to a faster electron transfer rate with its increase. Similar to the other material intrinsic activity, exf-TMD_small_ was the highest (0.21 mA/cm^2^) and TMD_mix_ was the lowest (0.13 mA/cm^2^). Turn over frequency (TOF) per surface metallic atom sites, estimated value at −150 mV vs RHE for exf-TMD_small_ was higher than for exf-TMD_big_ and TMD_mix_ (0.043, 0.024, and 0.005 H_2_/s respectively).

**Figure 5:**
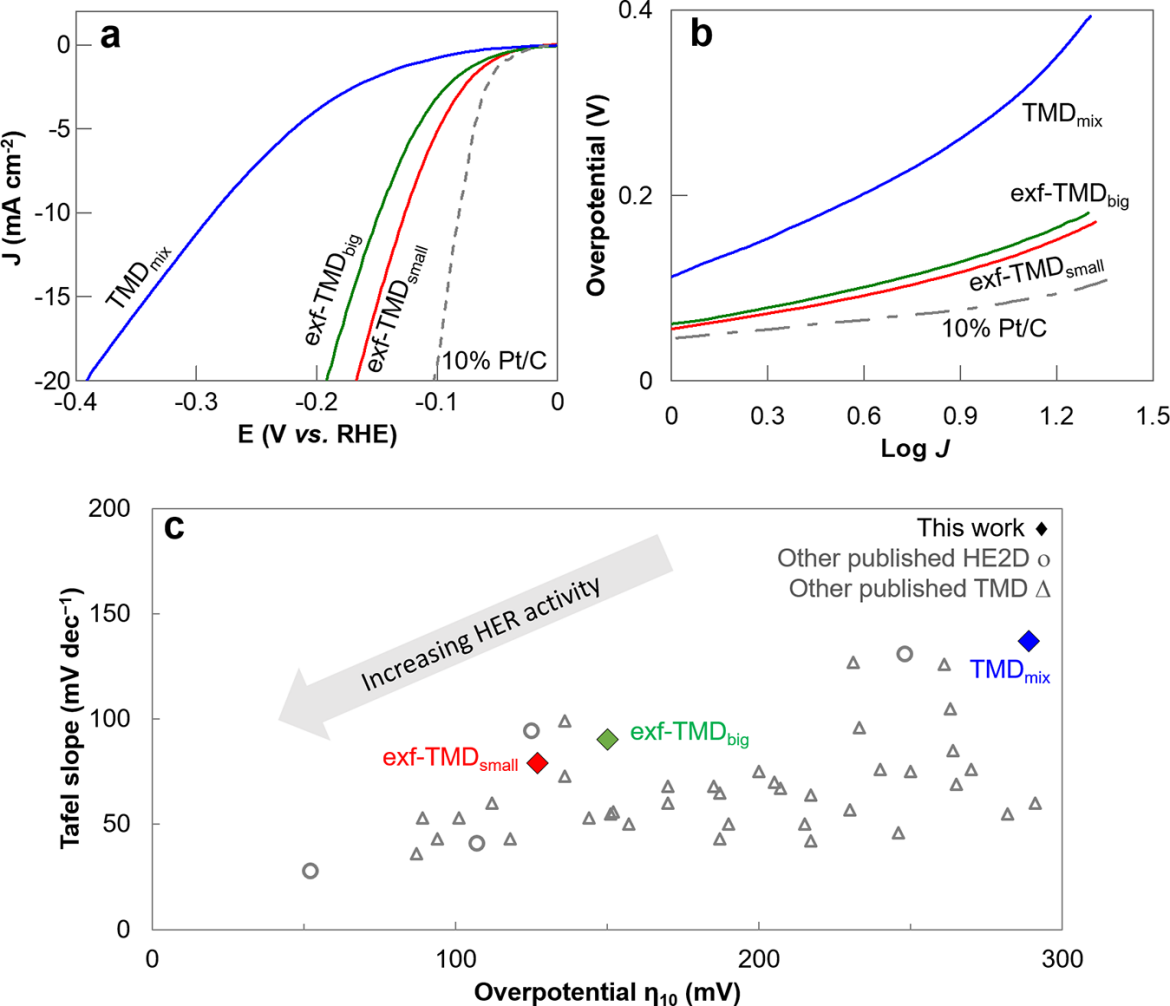
(a) Linear sweep voltammograms at 5 mV/s of materials in a cathodic potential window, in 0.5 M H_2_SO_4_. (b) Respective Tafel plots. (c) Distribution of Tafel slopes vs η_10_ the materials of this work and related published works with TMD and HE2D materials (values detailed in Table S2).

Details on the mass loading, η_10_, *j*_0_ and stability of materials in this work and the literature on TMDs and HE2D are shown for comparison in Table S3. As shown in Table S2, values in the literature can be found for η_10_ < 100 mV for HEA- or TMD-containing composites, which make these more competitive with the 10% Pt/C.^3,17,19–22,39–42^ Nevertheless, some cases do not clearly state the catalyst mass loading, and from the reports in Table S3, this parameter can range greatly from 0.04 to 167 mg/cm^2^, a critical aspect when comparing values across the literature since η_10_ is primarily affected by the loading mass of the material, leading to disparities in the number of active sites.^43^ The best value found in Table S3 is for np-HEA AlNiCuPtPdAu (with Al 97%), having a remarkable η_10_ of 52 mV and a TS of 28 mV/dec.^8^

To gain a better understanding of the HER activity trends for the TMD, the parameters of charge transfer resistance (*R*_ct_) and the nonfaradaic double-layer capacitance (*C*_dl_) were evaluated. The electrochemical impedance spectroscopy (EIS) was measured at −0.095 V vs RHE. The Nyquist plots are shown in Figure S8a, and the estimated *R*_ct_ fits the data in Figure S8b. The exf-TMD_small_ showed the lowest *R*_ct_ value of 118 Ω, indicative of a faster electron transfer process between the catalyst and the electrolyte. The CVs at different scan rates in the non-faradaic region used for determination of *C*_dl_ for TMD_mix_ material are shown in Figure S8c-e, by selection of the capacitive current as a function of the scan rates at the midpoint of the CVs. Plotting current density vs scan rate forms a linear relationship, where the slope is taken as *C*_dl_ (Figure S8f), which can be used to estimate the electrochemically active surface area (ECSA). The calculated *C*_dl_ increased from 0.02 mF/cm^2^ for TMD_mix_, up to 0.10 mF/cm^2^, which indicates that the exfoliated sample has a higher surface area of the material accessible to the electrolyte, also supported by the higher activity of the material toward HER.

For a more reliable activity measurement, additional tests were taken into account. First performing control of the η_10_ for 100 CVs, as shown in Figure S8g, in which exf-TMD_small_ had the lowest shift in the overpotential (Δη_10_). The ability of exf-TMD_small_ to reach a higher order of current densities and stability after 1000 CVs was also tested (Figure 6a). After multiple CVs, the Δη_10_ registered was ca. 68 mV, and the overpotential to reach −100 mA/cm^2^ (η_100_) was 390 mV, which increased to 480 mV, or a Δη_100_ of ca. 98 mV. Another strategy used in stability measurement is Δη_10_ in the galvanostatic method over time. The materials were tested for a short period of time, within 1 h, confirming that exf-TMD_small_ had the lowest η_10_ (Figure S8h). For exf-TMD_small_, the chronopotentiometry was extended to 24 h, with a Δη_10_ ca. 71 mV, or a decay of 2.9 mV/h (Figure 6b). An aliquot of the gas produced at the cathode was analyzed using gas chromatography with a thermal conductivity detector (GC-TCD), revealing a chromatographic profile similar to that of pure H_2_ gas (Figure S9). The evolved gas at −10 mA/cm^2^, for 1 h, was quantified by measurement of the gas collection volume from the cathode compartment. The generated quantities of produced gas amount to 0.353 ± 0.017 mmol H_2_, corresponding to a 94.7 ± 4.6% Faradaic efficiency (FE), when compared to the theoretical amounts of H_2_ (further details in SI).

**Figure 6:**
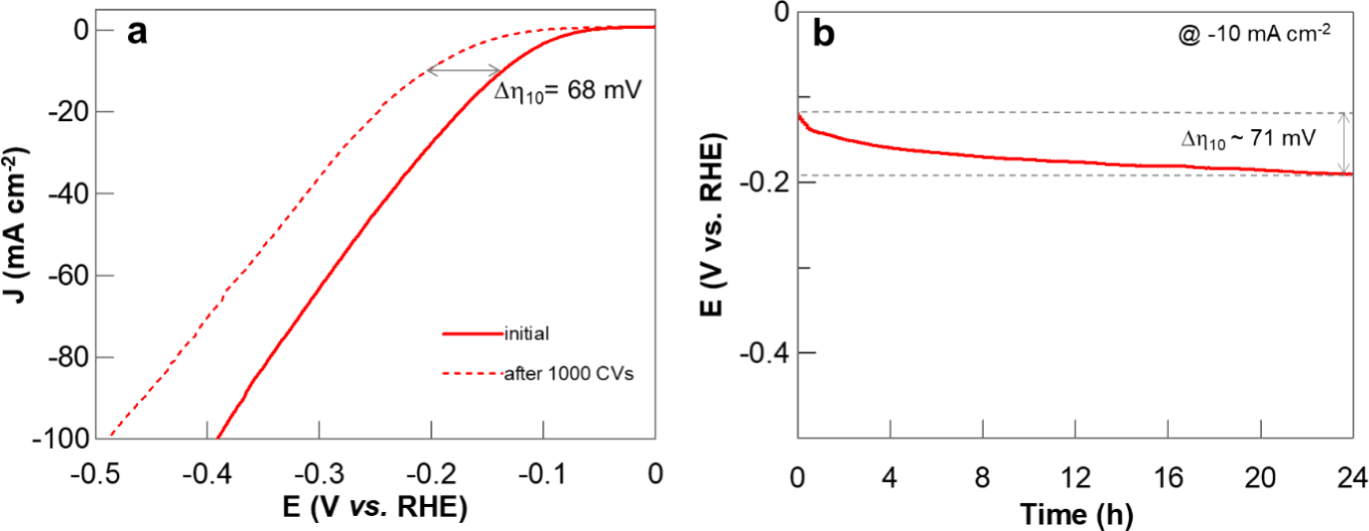
a) Linear sweep voltammograms at 5 mV/s of exf-TMD_small_ in the cathodic potential window, in 0.5 M H_2_SO_4_, before and after 1000 CVs, with cutoff at 100 mA/cm^2^. b) Stability of exf-TMD_small_ controlled by chronopotentiometry by applying current corresponding to −10 mA/cm^2^.

After long-term chronopotentiometry, the exf-TMD_small_ was characterized by STEM-EDX elemental mapping and XPS. The STEM-HAADF micrograph (Figure 7a) and EDX elemental mapping of individual sheets show some negligible changes in the shapes and composition of the sheets after catalysis, with all of the initial elements detected for the scanned sheets (Figure S10). The surface composition of exf-TMD_small_ postcatalysis was also characterized by XPS, which revealed only negligible changes in its chemical composition, confirming its good electrochemical stability (Figures 7b,c, and S11). The only noticeable difference was a slight broadening of some core-level peaks, which can be attributed to increased photoelectron scattering, likely due to surface modifications (different adsorbed species, mainly from electrolyte) rather than the formation of new chemical states. Importantly, no additional oxidation states or secondary phases emerged after the stability test. A small decrease in the Mo­(IV) B component, previously assigned to the trichalcogenide phase, was observed. As a result, the ratio of Mo­(IV) 3d (A) to Mo­(IV) 3d (B) changed from 5:1 to 7:1 (Figure 7c), indicating a slight redistribution of the bonding states while maintaining the overall structural and chemical integrity throughout long-term electrochemical testing. Figure S11a and b shows the spectrum regions corresponding to S 2p, S3 3p, and Te 3d, each with the component allocation and deconvolution.

**Figure 7:**
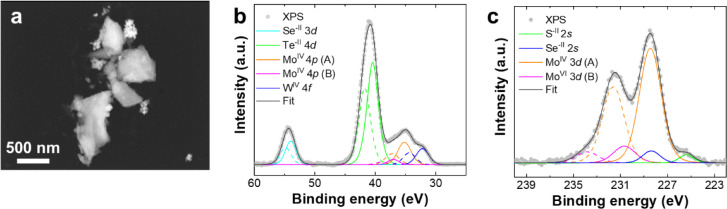
Postcatalysis characterization exf-TMD_small_. (a) STEM-HAADF micrograph of sheets after long-term HER measurement. Scale bar represents 500 nm. High resolution XPS spectrum for the range between (b) 60–25 eV and (c) 240–222 eV.

### 2.3 Computational Models

Based on the experimental results, the single crystalline 2D flakes used in this study are in 3R phase *a* = *b* = 0.318 nm, *c* = 1.337829 nm, α = β = 90°, γ = 120°, with the space group of *R*3*m* (160). The effect of material purity and atomic composition was deduced from EDX quantification. Based on the experimental composition, we constructed the model of Mo_0.5_W_0.5_(SSeTe)_2_, as shown in Figure 8a–c using VESTA. Figure 8d–f depicts the electronic band structures of MoS_2_, Mo_0.5_W_0.5_(SSeTe)_2_, and Mo_0.5_W_0.5_(SSeTe)_2_ with hydrogen atoms adsorbed at a surface site; the band structures are calculated along the high-symmetry points in the Brillouin zone (Γ, K, M).

**Figure 8:**
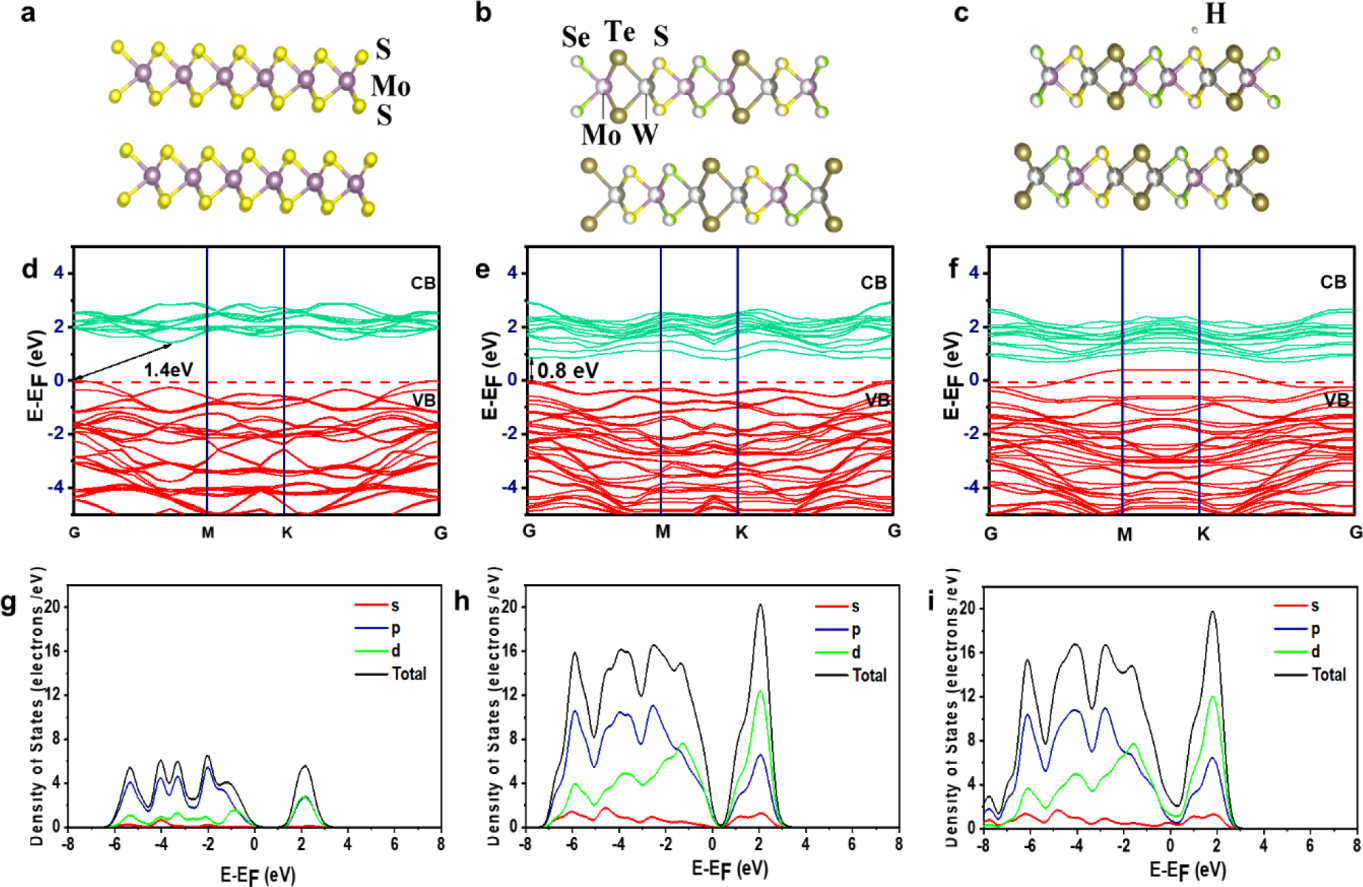
(a–c) MoS_2_, Mo_0.5_W_0.5_(SSeTe)_2_, and Mo_0.5_W_0.5_(SSeTe)_2_ with H atoms adsorbed modeled structure. (d–f) Electronic band structure of MoS_2_, Mo_0.5_W_0.5_(SSeTe)_2_, and Mo_0.5_W_0.5_(SSeTe)_2_ with H atoms adsorbed. (g–i) Partial and total densities of states of MoS_2_, Mo_0.5_W_0.5_(SSeTe)_2_, and Mo_0.5_W_0.5_(SSeTe)_2_ with H atoms adsorbed.

The top of the valence band maximum (VBM) and the bottom of the conduction band minimum (CBM) do not overlap and diverge at the Brillouin zone (BZ) symmetry point responsible for the indirect–direct band gap, *E*_g_ is 1.4 eV, validating with previously reported results for MoS_2_.^44^ In the case of Mo_0.5_W_0.5_(SSeTe)_2_, the band gap changes to be a direct transition band gap of 0.8 eV at the Gamma-point. When Mo_0.5_W_0.5_(SSeTe)_2_ with H atoms is adsorbed at the Γ-point, the band structure remains unaffected by hydrogen adsorption. However, at the *K* and *M* points, hydrogen adsorption induces noticeable changes in the electronic properties. Specifically, the valence band energy increases at these points, surpassing the Fermi level, while the energy gap at the Γ-point remains unchanged.

The valence and conduction band electron contributions can be well understood with the density of states calculations. The total density of states (TDOS) and partial density of states (PDOS) for MoS_2_, Mo_0.5_W_0.5_(SSeTe)_2_, and Mo_0.5_W_0.5_(SSeTe)_2_ with H atoms are shown in Figure 8g–i. The pseudo-atomic calculations of the valence electrons of S 3s^2^ 3p^4^, Se 4s^2^ 4p^4^, Mo 4s^2^ 4p^6^ 4d^5^ 5s^1^, Te 5s^2^ 5p^4^ and W 5s^2^ 5p^6^ 5d^4^ 6s^2^ were performed to understand the density of states of the system for Mo_0.5_W_0.5_(S Se Te)_2_. Figure S12a-c shows the optimized structures of the pristine MoS_2_ surface slab, Mo_0.5_W_0.5_(SSeTe)_2_, and Mo_0.5_W_0.5_(SSeTe)_2_ with H atoms adsorbed at the unique surface site.

The results show that the electronic density of the adsorbed H strongly overlaps with that of the modified Mo_0.5_W_0.5_(SSeTe)_2_ slab model, indicative of a strong interaction. The strong overlap in the total electronic density of the adsorption system is a typical signal revealing a chemical interaction between the adsorbed H and Mo_0.5_W_0.5_(SSeTe)_2_. Meanwhile, the PDOS of the adsorbed H-1s^1^ orbital with the W 5d^4^ and S 3p^4^ hybridized strongly near the Fermi level, as shown in Figure 8i.

Interestingly, the PDOS of the H overlapped with the S–P orbital of H and S causes the overlap of the valence band above the Fermi level in the band structure at the *M* and *K* points, as can be seen in Figure 8f which plays a major role in determining the adsorption of H. The dominance of sulfur arises due to its relatively stronger interaction with the W 5d orbitals and the adsorbed hydrogen 1s orbital. This is evident from the PDOS analysis, where sulfur exhibits significant hybridization near the Fermi level, contributing prominently to the electronic states. In contrast, the contributions from Se and Te are less pronounced in this energy range. Furthermore, the absence of similar quantum states in pristine MoS_2_ (as shown in Figure 8g) highlights the role of alloying and hydrogen adsorption in modifying the electronic structure. The interaction between the S p-orbitals, W d-orbitals, and adsorbed hydrogen introduces new states near the Fermi level, enhancing the material’s catalytic properties for hydrogen evolution reactions.

The adsorption energy of H on Mo_0.5_W_0.5_(SSeTe)_2_ is calculated from *E*_ads_ = *E*_total_ – (*E*_t_ + *E*_h_), where *E*_ads_, *E*_total_, *E*_g_, and *E*_h_ denote the adsorption energy, total energy of Mo_0.5_W_0.5_(SSeTe)_2_-H, ground-state energy of Mo_0.5_W_0.5_(S Se Te)_2_, and H, respectively. It is reported that the large *E*_ads_ value of 1.61 eV for pristine MoS_2_,^4^ results in slow H binding and poor HER activity. In contrast, for Mo_0.5_W_0.5_(S Se Te)_2_-H, we observed *E*_ads_ is −1.54 eV, indicating higher adsorption energy at the unique surface site, which can increase the good HER activity.

## 3 Conclusions

In summary, we have explored a 2D material and entropy mixture of TMD applied to the synthesis of Mo_0.5_W_0.5_(SSeTe)_2_ by the CVT method. The estimated stoichiometry of the TMD entropy mixture (TMD_mix_) was estimated as Mo_0.56_W_0.44_(S_0.33_Se_0.35_Te_0.32_)_2_ and a theoretical Δ*S*_mix_ estimated as 0.96*R*. The 2D flakes have a crystalline ordered structure, evidenced by the atomic arrangement patterns, which indicates the presence of different phases of TMDs (1T, 2H, and 3R). The exfoliated material (exf-TMD_small_) was the best catalysts with a low η_10_ of 127 mV, η_100_ of 390 mV, and a Tafel slope of 79 mV/dec, lowest *R*_ct_, highest TOF, MA, and *C*_dl_. At a current density of −10 mA/cm^2^, the electrocatalytic performance of exf-TMD_small_ is sustained for a duration of 24 h, although there was a decay in the material activity of 2.9 mV/h. Postcatalysis characterization indicates minimal shape and elemental loss, attributed to the sluggish diffusion and lattice distortion effects, preventing transition metals from leaching and dissolution. Computational studies indicate that, in the case of Mo_0.5_W_0.5_(SSeTe)_2_, the band gap changes to a direct transition band gap of 0.8 eV at the Γ-point, having also a more favored H adsorption energy, which could be the main reason the small flakes of Mo_0.5_W_0.5_(SSeTe)_2_ showed an improved HER response.

## 4 Experimental Section and Methods

### 4.1 Chemicals

Sulfur (99.999%, granules), selenium (99.999%, granules), tellurium (99.999%, granules), molybdenum, and tungsten (metallic powders, purity 99.9%, 3 and 12 μm grain sizes, respectively) were acquired from STREM, Germany. Potassium hydroxide (KOH), 5 wt % Nafion perfluorinated resin solution, and platinum nanoparticles supported on carbon with 10 wt % loading (10% Pt/C) were purchased from Sigma-Aldrich, Czech Republic. Ethanol (EtOH) was purchased from Penta, Czech Republic.

### 4.2 Synthesis of Materials and Chemical Exfoliation

The bulk TMD_mix_ was prepared from the corresponding elemental powders in sealed quartz glass ampules. Stoichiometric amounts of Mo,W, S, Se, and Te in order to reach MX_2_ in quantities up to 10 g of TMD were placed in a quartz glass ampule (180 × 25 mm; 2 mm wall thickness), with iodine (0.4 g) as the transport agent and evacuated at a base pressure of 1 mPa. Subsequently, the quartz ampule was sealed with an oxygen–hydrogen welding torch. The elements were heated up to 600 °C for 48 h. The ampule contents were mechanically mixed for 10 min, followed by heating to 800 °C for 48 h, and then to 850 °C for 12 h after the first heating procedure. All heating and cooling rates were 5 °C/min. After the formation of dichalcogenide, the ampule is placed in a two-zone furnace for chemical vapor transport growth. First, the reverse thermal gradient was applied to clean the growth part of the ampule. The growth zone was heated to 600 °C and the source zone to 900 °C for 2 days. Subsequently, the thermal gradient was reversed, and the temperature of the growth zone was set to 800 °C and the source temperature at 900 °C for 7 days. Finally, the furnace was freely cooled at room temperature, and the ampule was opened in the glovebox to obtain the crystals.

The exfoliation of the wide crystals of the starting TMD_mix_ was carried out by suspending 1 g of the material powder in 20 mL of 1.6 M *n*-butyllithium in hexane. The slurry was stirred for 72 h at 25 °C under an argon atmosphere. The Li-intercalated material was then separated by vacuum filtration, and the intercalation compound was washed several times with *n*-hexane. The separated material with intercalated Li was resuspended in water and centrifuged. In the last centrifugation cycle, the material was separated into the bottom phase (exf-TMD_big_) and the top phase (exf-TMD_small_). The obtained material was dried in a vacuum oven at 50 °C for 48 h prior to further use.

### 4.3 Structural and Morphological Characterization

Synthesized materials were analyzed by using various techniques: digital imaging with an optical profilometer (Sensofar), bulk morphology with SEM (Tescan Maia, at 5 kV), and exfoliated material morphology with STEM (Tescan Maia, at 20 kV). Elemental composition was studied by using EDX (X-MaxN, at 20 kV). Structural analysis was performed with XRD (Bruker D8, Cu Kα radiation), while Raman spectroscopy was performed using a Renishaw inVia microscope with a 532 nm Nd:YAG laser. TEM (JEOL ARM200F, 200 kV) provided high-resolution imaging with monochromators, correctors, and EDX, EELS, and HAADF detectors. Surface composition was further examined by XPS (SPECS, Al Kα, Phoibos 150), and sample composition was obtained by inductively coupled plasma optical emission spectrometry (ICP-OES). Measurements were performed with a Spectro ARCOS (SPECTRO Analytical Instruments). Further details on experimental conditions are given in the SI.

### 4.4 Electrochemical Measurements

These experiments were conducted at room temperature in a three-electrode configuration using an Autolab PGSTAT204 instrument (Eco Chemie, Utrecht, The Netherlands) controlled by NOVA Version 2.1.7 software. A glassy carbon (GC) electrode (3 mm diameter) was used as the working electrode (WE), a carbon rod was used as the counter electrode, and Ag/AgCl/KCl sat. as the reference electrode (RE), unless another electrode is specified. All electrodes were acquired from CH Instruments, Texas, USA. Each TMD material was dispersed in H_2_O at a concentration of ca. 5 mg mL^–1^ and initially sonicated for 10 min in an ultrasonic bath (*T* < 20 °C, 37 Hz, 100%). The WE modification was done by drop-casting each suspension, corresponding to an average loading of 0.7–0.9 mg cm^–2^, on the WE surface to form a thin film. On top of the material film, 1 μL of 1% Nafion solution was drop cast. All the experiments were carried out in purged 25 mL of 0.5 M H_2_SO_4_ or 1.0 M KOH electrolyte and are reported without *i*R compensation. The HER experiments were carried out by linear sweep voltammetry (LSV) at a scan rate of 5 mV s^–1^ vs RE. All potentials were converted to the reversible hydrogen electrode (RHE) according to the Nernst equation: *E*_vs RHE_ (V) = *E*_vsRE_ + 0.059 pH + *E* ^0^_Δ(RHE‑RE)_. Chronopotentiometry measurements were done at a fixed current of −10 mA cm^–2^ for 1 or 24 h. Further details on the calculations and equations are given in the Supporting Information.

### 4.5 Theoretical Investigation

Based on the experimental results, the single crystalline 2D flakes used in this study are of the mixed metal MoS_2_ 3R phase *a* = *b* = 0.318 nm, *c* = 1.337829 nm, α = β = 90°, γ = 120°, with the space group of *R*3*m* (160). Based on the experimental composition, we constructed a slab model with two layers of Mo_0.5_W_0.5_(SSeTe)_2_ along with the pristine 3R-MoS_2_ surface, and Mo_0.5_W_0.5_(SSeTe)_2_ with H atoms adsorbed at the unique surface site was modeled using VESTA. The density functional theory simulation of the identified 2D structure, involving properties such as structure optimization, band structure, and density of states (DOS), was calculated using Material Studio-CASTEP. For the structural optimization, the ultrasoft pseudopotential was used to simulate the interaction between electrons and ion cores in geometric structure optimization and single-point energy calculation, and the PBE (Perdew–Burke–Ernzerhof) in generalized gradient approximation (GGA) was used to describe the exchange-correlation function. The scissors value is used to correct the calculated band gap value. The cutoff energy was set to 350 eV, and the *K*-point was set to 4 × 2 × 2. The convergence threshold for SCF tolerance is 2 × 10^–6^ eV/atom between two electronic steps, and the maximum force upon each atom is less than 0.01 eV/Å.

## Supplementary Material



## Data Availability

The data sets generated during and/or analyzed during the study are accessible via the Zenodo repository: 10.5281/zenodo.13271038.

## Notes

Additional Notes on characterization techniques and electrochemical methods. Supporting TEM, SEM and STEM micrographs, EDX elemental maps and spectra; XPS deconvolution of the spectrum. EIS, double-layer capacitance plots, electrochemical stability of materials; GC-TCD patterns of evolved gas; ICP-OES table; supporting data of computational modeling (PDF); EDX table; Equations.
