# Supplementary material for: Toward 2D van der Waals Entropy Mixture MX2 (M = Mo, W; X = S, Se, Te) for Hydrogen Evolution Electrocatalysis
Source: ACS Appl Mater Interfaces. 2025 Jun 4;17(24):35522–32. doi: 10.1021/acsami.5c05482 (PMC12186216; doi:10.1021/acsami.5c05482)
Supplement: Supplementary file 1 [file am5c05482_si_001.pdf]

SUPPORTING INFORMATION

Towards 2D van der Waals Entropy Mixture MX_2 (M=Mo,W; X=S,Se,Te) for Hydrogen Evolution Electrocatalysis

Jan Paštika,[a] Deniz Güngen,[a] Amutha Subramani,[a] Vlastimil Mazánek,[a] Marco Serra,[a,b] Lunjie Zeng,[c] Eva Olsson,[c] Rui Gusmão[a] and Zdeněk Sofer[a]***

[a] Department of Inorganic Chemistry, Faculty of Chemical Technology, University of Chemistry and Technology Prague, Technická 5 Prague 6, 166 28, Czech Republic

[b] Present address: Istituto Italiano di Tecnologia, Via Morego 30, 16163 Genova, Italy

[c] Department of Physics, Chalmers University of Technology, Gothenburg SE-41296, Sweden

*   rui.gusmao@vscht.cz

** zdenek.sofer@vscht.cz

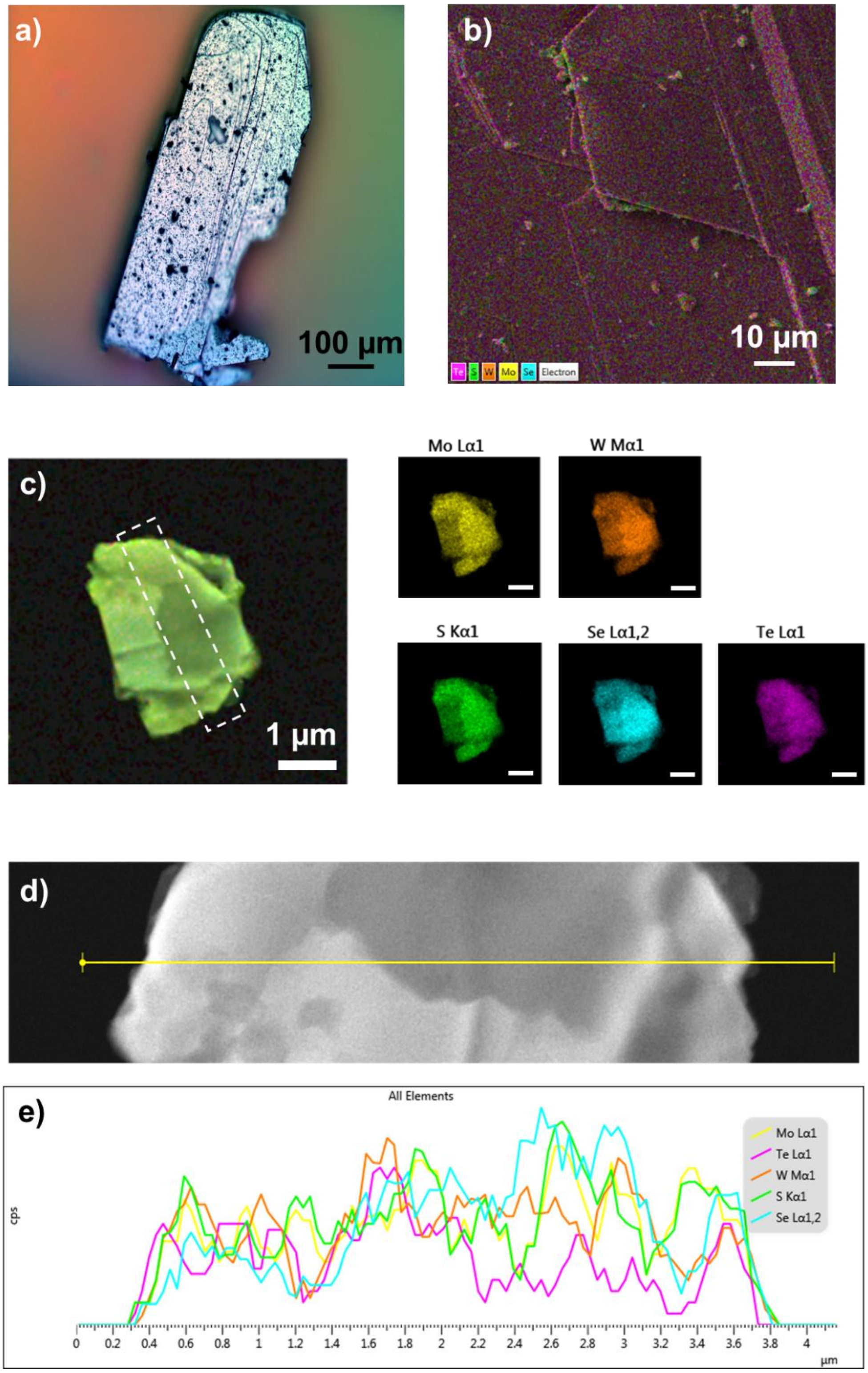

Figure S1. a) Optical image of TMD_{mix} crystal, scale bar indicates 100 μm. b) Overlayered SEM micrograph with elemental mapping c) Dark field mode STEM electronic image with respective mapping of elements. d) Location of selected area from flake for EDX line scan analysis. e) Respective EDX composition along the line scan with CPS for Mo Lα1, W Mα1, S Kα1, Se Lα1,2 and Te Lα1.

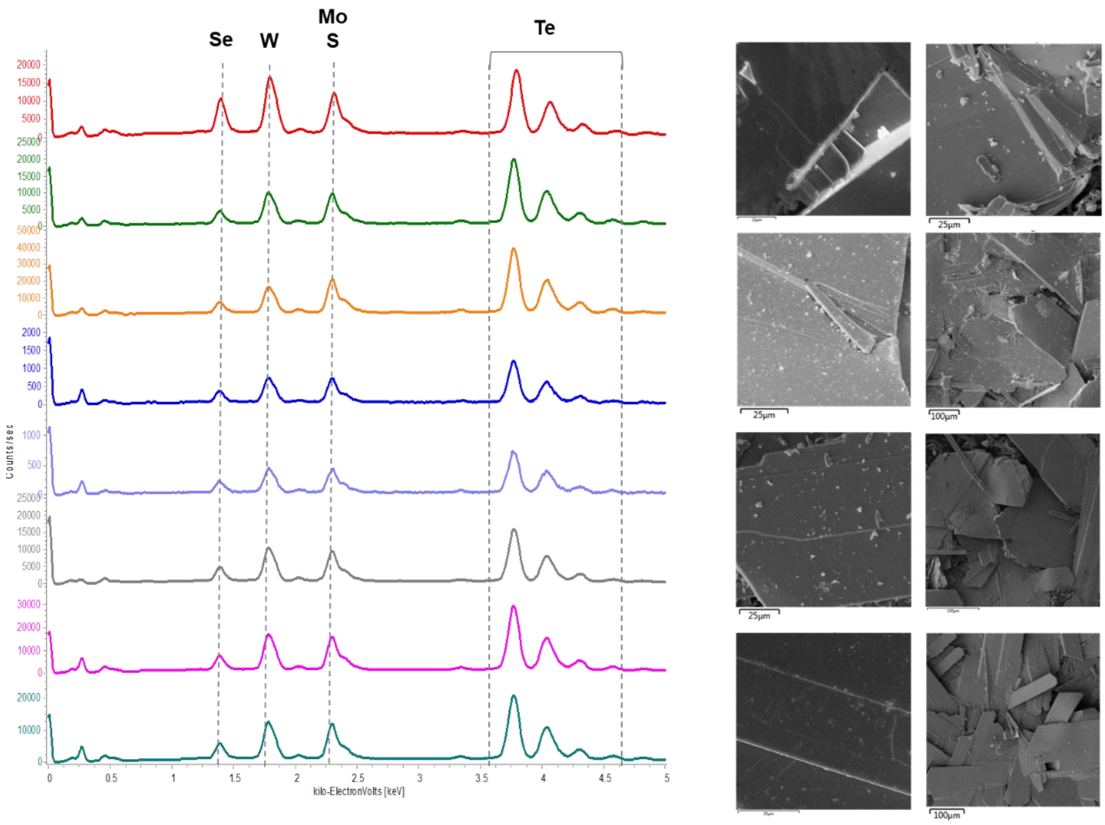

Figure S2. EDX spectra of TMD_{mix} crystals and corresponding SEM areas used for their acquisition, demonstrating elemental distribution and characteristic layered crystalline morphology with platelet-like structures (scale bars: 10-100 μm).

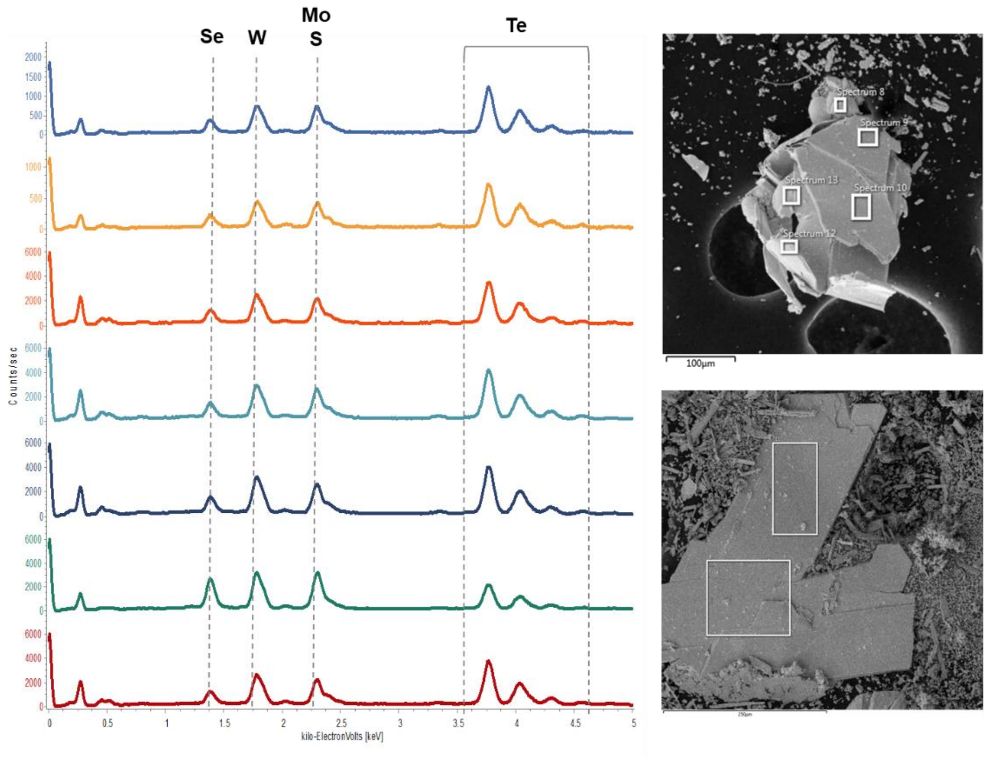

Figure S3. Additional EDX spectra of TMD_{mix} crystals and corresponding SEM/EDX Point&ID areas used for their acquisition (scale bars: 100 and 250 μm).

STEM image simulation: STEM image simulations for the 1T, 2H, and 3R phases of TMDs were carried out using the µSTEM program.[1] Structure models for the phases were obtained from the ICSD database. The simulations were performed using the multi-slice method by adopting an absorptive potential model and taking absorption into account. The sample thickness was kept at 10 nm. Electron beam energy was 200kV. Experimental beam and detector settings were used.

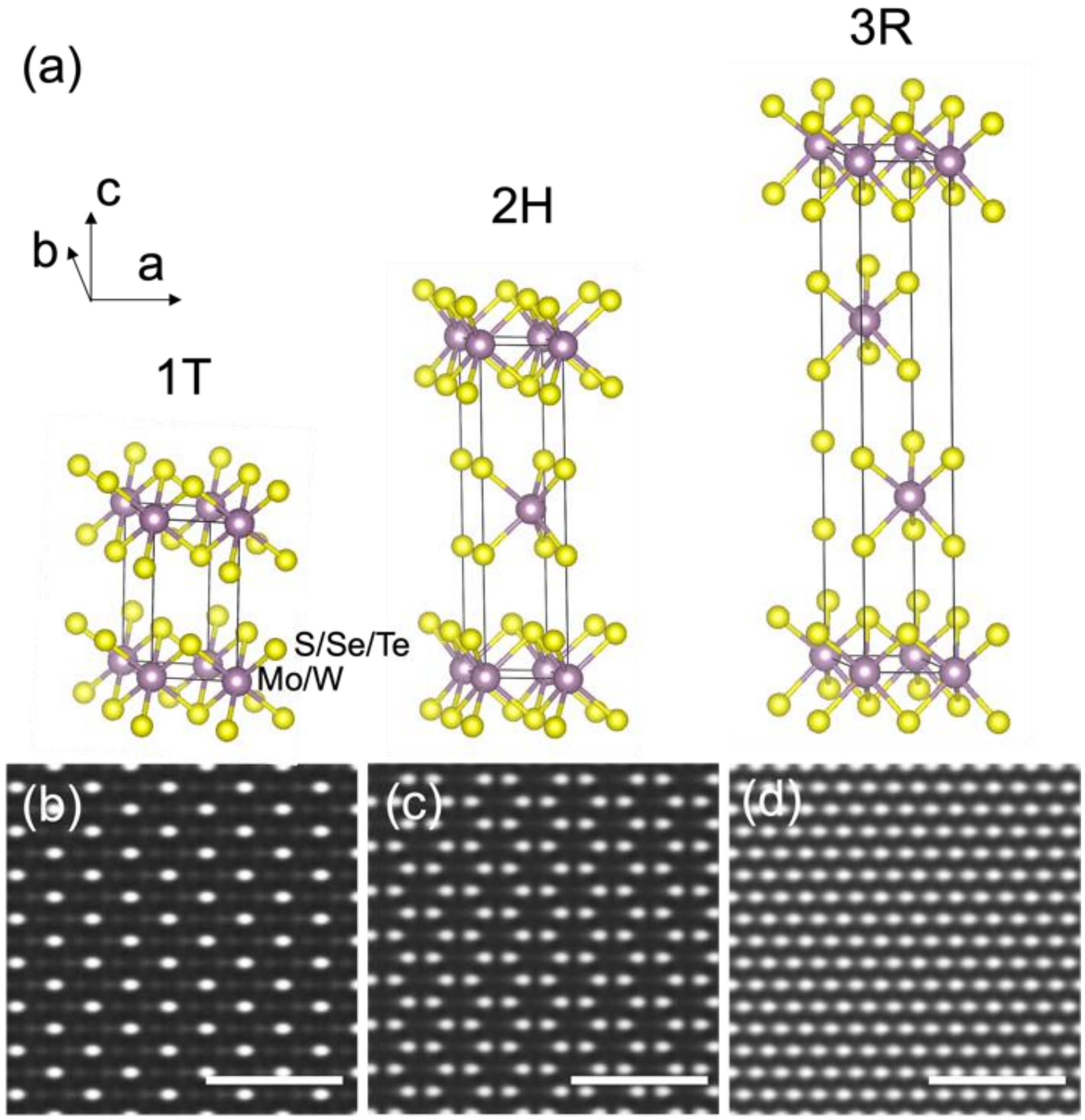

Figure S4. (a) Structure models of the commonly found 1T, 2H, and 3R phases of TMDs. (b)-(d) Simulated [001] zone-axis STEM HAADF images of the 1T, 2H and 3R phases of $MoTe_2$, respectively. The scale bars are 1 nm.

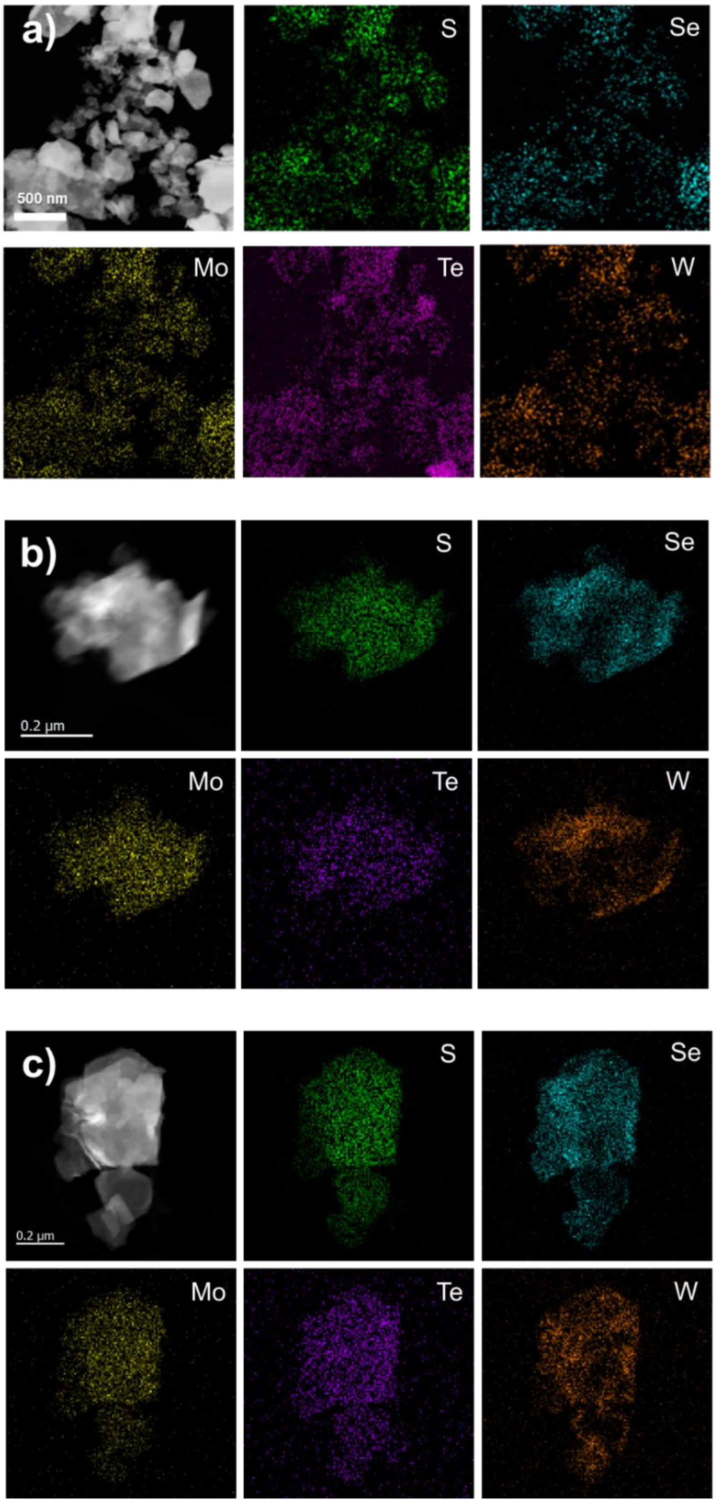

Figure S5. Dark field STEM of TMD_{mix} (a), and STEM HAADF images of TMD_{mix} flakes (b and c). Respective elemental maps of S, Se, Mo, Te, and W. Scale bars correspond to 500 and 200 nm.

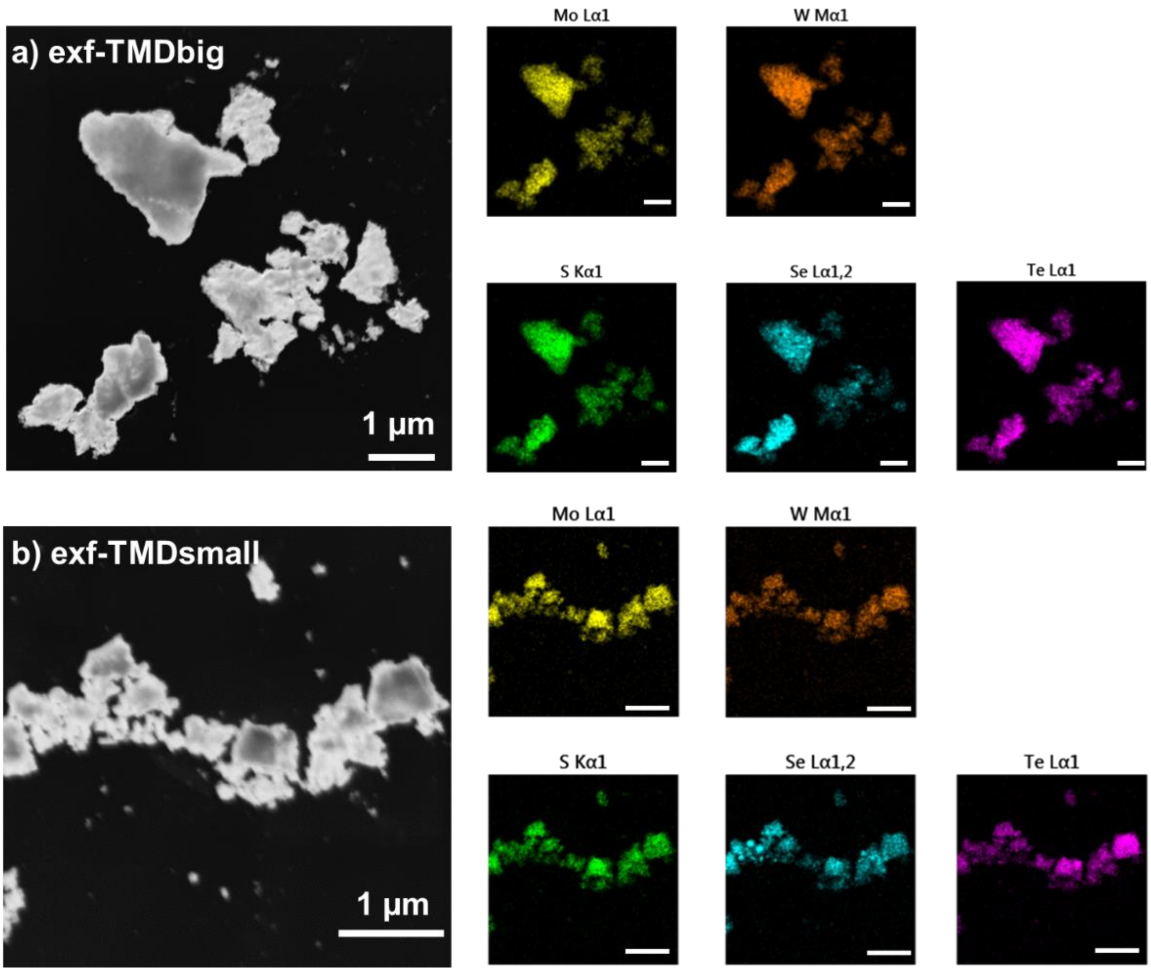

Figure S6. Dark field STEM of exf-TMD_{big} (a) and exf-TMD_{small} (b) flakes produced from chemical exfoliation. Scale bar: 1 µm. Respective elemental maps of S, Se, Mo, Te, and W.

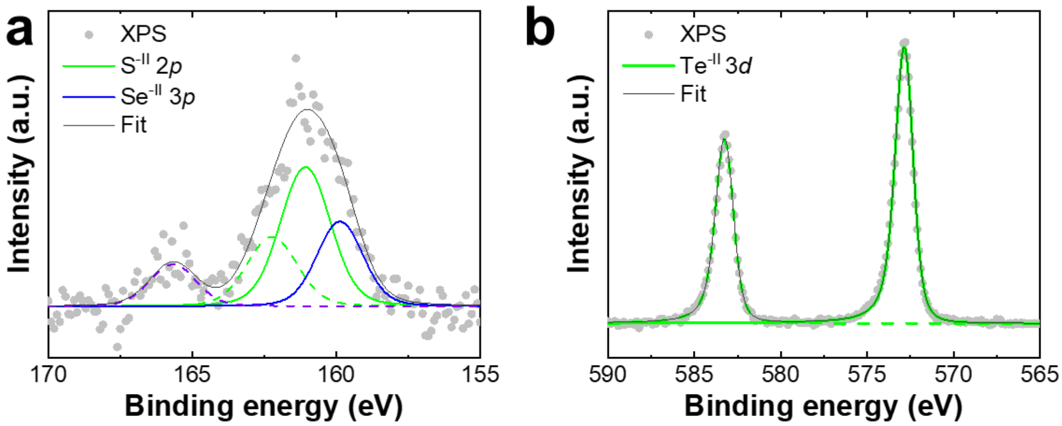

Figure S7. High resolution XPS spectrum for the range between a) 170-155 corresponding to S 2*p* and Se 3*p* eV, and b) 590-565 eV related to Te 3*d*.

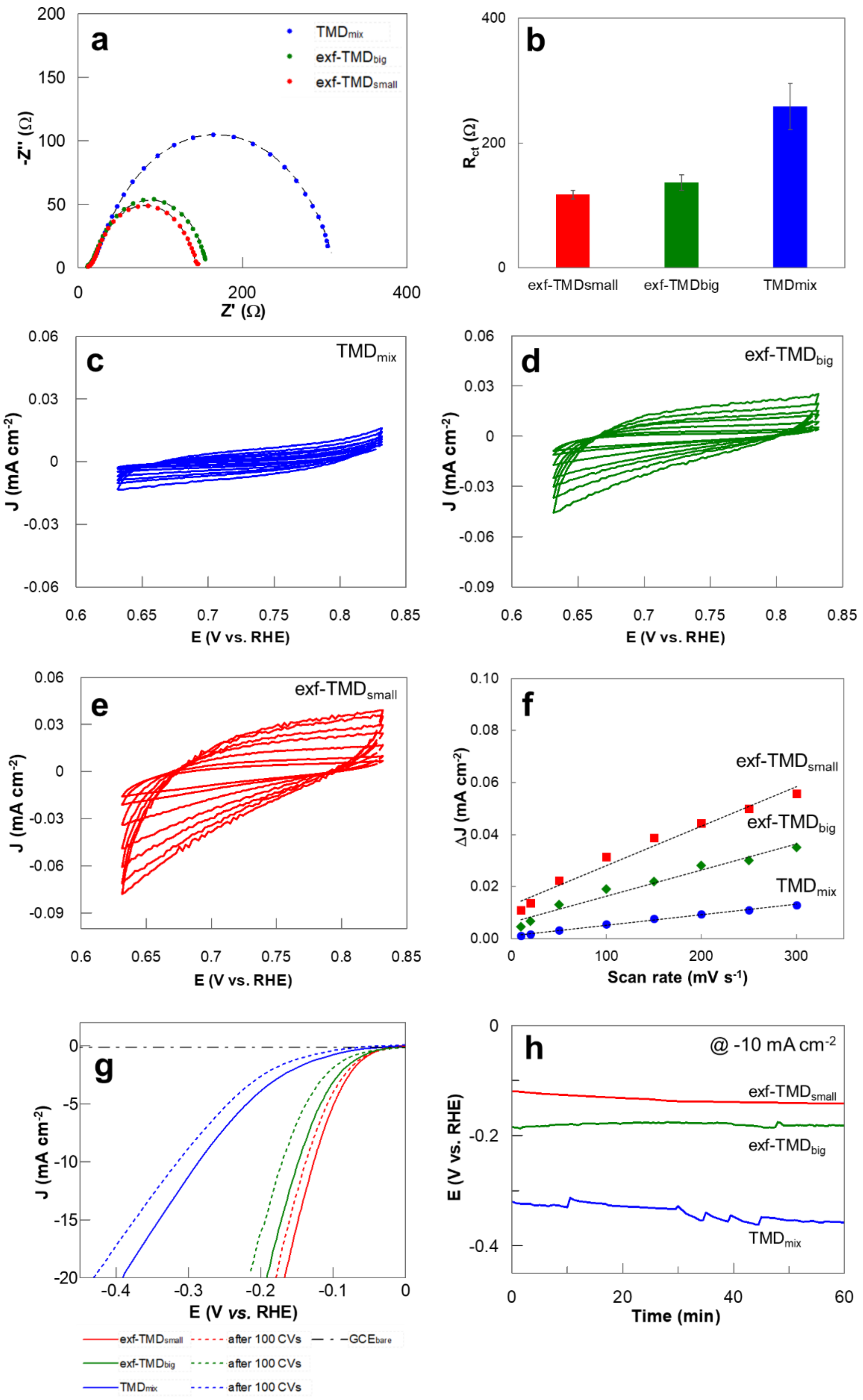

Figure S8. (a) Nyquist plots at −0.095 V vs. RHE. (b) Respective estimated R_{ct}. Anodic scan cyclic voltammograms of (c) TMD_{mix} (d) exf-TMD_{big} and (e) exf-TMD_{small} at different scan rates (ν = 10, 20, 50, 100, 150, 200, 250, 300 mV/s). (f) Determination of double layer capacitance (C_{dl}) from respective CVs at +0.723 V versus RHE. (g) First and 100th CV of the materials at 5 mV/s. h) One hour chronopotentiometry of the materials at density current to -10 mA/cm^2.

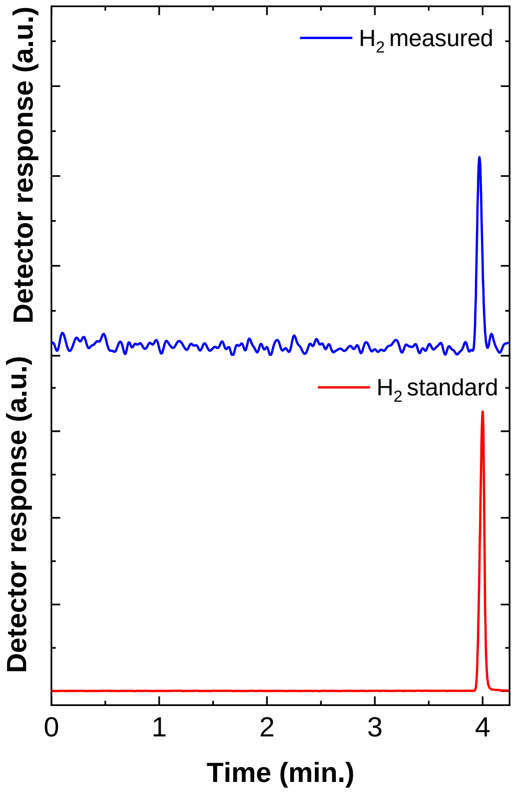

Figure S9. GC-TCD patterns of evolved gas during HER at cathode and H_2 standard gas.

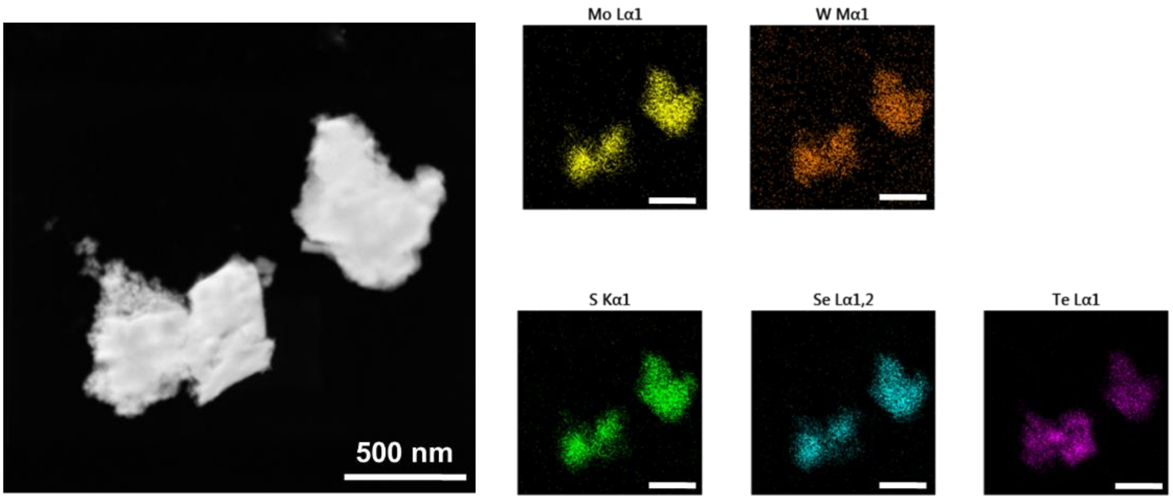

Figure S10. EDX of the exf-TMD$_{small}$ sheets after long HER measurement. Dark mode STEM micrograph and respective mapping of elements for exf-TMD$_{small}$ sheets extracted from the film at GC surface after electrochemistry. Scale bars represent 500 nm.

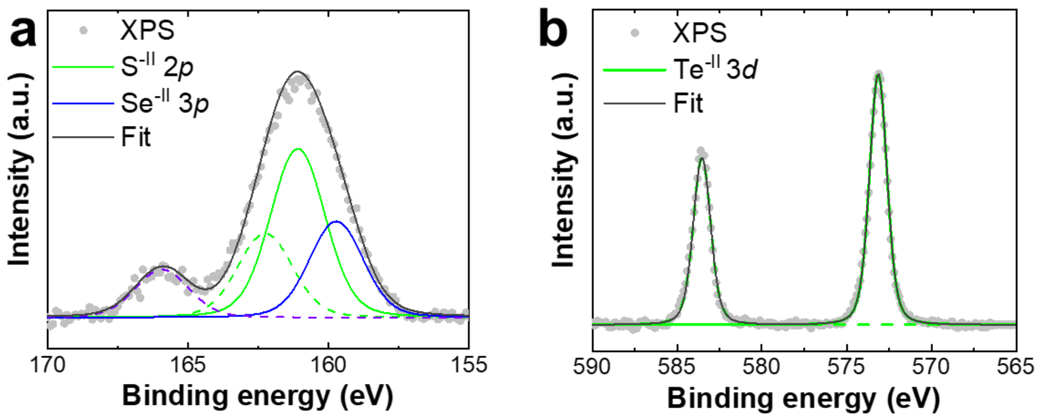

Figure S11. High resolution XPS spectra of exf-TMD_{small} after long HER measurement: a) 170-155 corresponding to S 2*p* and Se 3*p* eV, and b) 590-565 eV related to Te 3*d*.

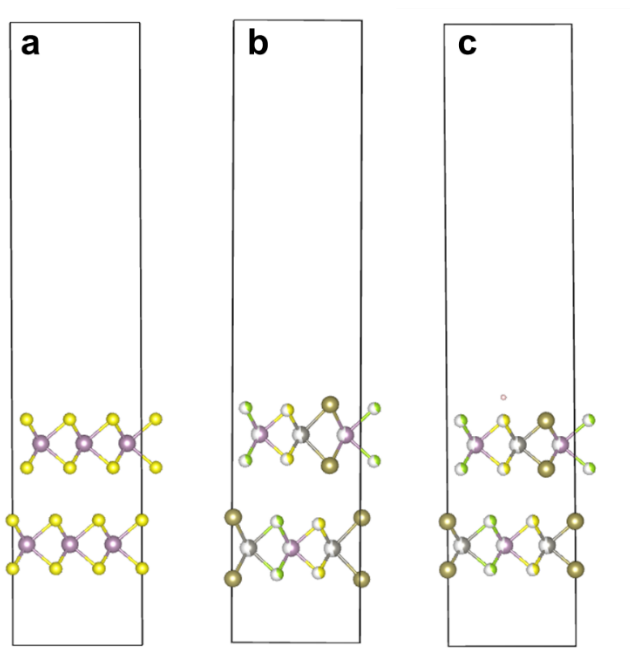

Figure S12. a) Optimized pristine MoS_2 surface slab b) Optimized slab $Mo_{0.5}W_{0.5}(SSeTe)_2$ c) Optimized slab $Mo_{0.5}W_{0.5}(SSeTe)_2$ with H atoms adsorbed at the unique surface site. These images were prepared using the VESTA software.

Table S1. Average at.% of elements and respective standard deviation for the TMD_{mix} synthetized from EDX quantification. Average was obtained from the different spots shown in Figures S1b, S2 and S3.

**Element**	**Mo**	**W**	**S**	**Se**	**Te**
**Avg. at.%**	20.5	16.5	5.2	6.5	51.2
**σ**	3.8	3.5	5.3	4.0	8.3

Table S2. Composition of TMD_{mix} wt.% of elements obtained by ICP-OES.

Element	Mo	W	S	Se	Te
**wt. %**	17.7	27.0	7.4	18.8	26.9

Table S3. Figures of merit and results obtained for this work in comparison with published reports of HE2D materials and TMDs for HER in 0.5 M H_2SO_4.

Material	Loading [mg/cm^2]	η_{10} [mV]	Tafel slope [mV/ dec]	j_0 [mA/cm^2]	Stability	Ref.
exf-TMD_{small}	0.75	127	80	19.3	24 h + 1000 cycles	this work
exf-TMD_{big}	0.92	169	89	10.3	N/A	this work
TMD_{mix}	0.80	293	142	6.6	N/A	this work
np-HEA ($Al_{97.5}Ni_{0.5}Cu_{0.5}Pt_{0.5}Pd_{0.5}Au_{0.5}$)	0.42	52	28	N/A	2000 cycles	[2]
WSe_2/Sn	50	87	36	580	5000 cycles	[3]
MoS_2/$Co(OH)_2$	0.20	89	53	73	20 h	[4]
TiO_2/Si/$MoSe_2$	167	94	43	N/A	10 h	[5]
TaS_2@Au	N/A	101	53	N/A	12 h	[6]
$Ni_{20}Fe_{20}Mo_{10}Co_{35}Cr_{15}$	N/A	107	41	N/A	8 h	[7]
WS_2	0.10	118	43	21	30 h	[8]
$CoVMnNiZnPS_3$	0.35	125	94.5	0.4	3000 cycles	[9]
MoS_2@Au	N/A	136	73	0.01	200 h	[10]
MoS_2@ porous C	1.00	136	99	76	24 h	[11]
TiP_2S_6@$MoTe_2$	13	144	53	N/A	4.2 h	[12]
MoS_2@carbon fiber	17	151	55	N/A	1000 cycles	[13]
1T-$MoSe_2$	0.1	152	56	N/A	1000 cycles	[14]
Pd_xNbS_2	0.25	157	50	N/A	12 h	[15]
Ni_3S_2/FeS/CoS	N/A	170	68	N/A	50 h	[16]
1T-WS_2	0.1	170	60	N/A	10000 cycles	[17]
Co/MoS_2	0.27	185	68	68	11 h	[18]
1T-MoS_2	0.1	187	43	8.9×10^{-3}	1000 cycles	[19]
Porous MoS_2 nanosheets	0.29	190	50	N/A	5000 cycles	[20]
WS_2	N/A	205	70	N/A	N/A	[21]
TaS_2@Au	N/A	207	67	68	24 h	[22]
MoS_2@C_3N_4	0.28	215	50	80	N/A	[23]
WSe_2/$Co_{0.85}Se$	N/A	217	64	N/A	N/A	[24]
$CoTe_2$@carbon paper	4.85	230	57	0.01	5000 cycles	[25]
$MoTe_2$@carbon cloth	N/A	231	127	N/A	1000 cycles	[26]
Co_9S_8/MoS_2@NSOC	N/A	233	96	N/A	12 h	[27]
MoS_2	N/A	240	76	N/A	N/A	[28]
$CoTe_2$	N/A	246	46	N/A	N/A	[29]
(AgCuZnMnCoInGa)S@20%CB	0.60	248	131	N/A	7000 cycles + 20 h	[30]
Co_9S_8/NC@MoS_2	N/A	261	126	N/A	12 h	[31]

Vertically aligned $MoSe_2$	0.28	263	105	2.2×10^{-3}	10000 cycles	[32]
$ReSe_2$	N/A	265	69	N/A	2.7 h	[33]
$ReSe_2$@SiO_2/Si	N/A	270	76	0.01	1000 cycles	[34]
MoS_2 nanoparticles	0.70	282	55	N/A	10 h	[35]
MoS_2 NDs/VS_2	N/A	291	60	N/A	16 h	[36]
WSe_2@carbon paper	0.04	301	150	N/A	N/A	[37]
$NiTe_2$@Ti mesh	0.95	315	82	N/A	24 h	[38]
$MoTe_2$	N/A	356	22	0.02	2000 cycles	[39]

Additional Notes

Characterization Techniques

Digital photographs of the synthesized crystals were taken with a Sensofar optical profilometer microscope. The morphology of the bulk materials was examined by scanning electron microscopy (SEM) with a FEG electron source (Tescan Maia dual-beam microscope) with a 5 kV acceleration voltage. The morphology of exfoliated materials was observed by scanning transmission electron microscopy (STEM) with a 20 kV acceleration voltage. The elemental composition of bulk and exfoliated materials was investigated by EDX using an X-Max^N detector from Oxford Instruments with a 20 kV acceleration voltage. Samples of bulk materials were directly placed on carbon tape; concerning exfoliated materials, suspensions were prepared, and thus for each sample 5 µL were dropped cast on STEM grids (from TED PELLA, Inc.) and dried at room temperature. C and O were detected in the EDX spectra from the underlying carbon tape used as support and partial surface oxidation during manipulation.

XRD was done with a Bruker D8 Discoverer diffractometer in Bragg–Brentano parafocusing geometry and using a CuKα radiation source (λ = 0.15418 nm, U = 40 kV and I = 40 mA), and the XRD patterns were collected for 2θ values from 10° to 70° at room temperature. The bulk crystals were harshly ground to a powder for XRD measurements. The collected data were analyzed by using HighScore Plus 4.9 software, and experimental patterns were confirmed on Inorganic Crystal Structure Database (ICSD). An inVia Raman microscope (Renishaw, England) was used for Raman spectroscopy measurements in backscattering geometry with a CCD detector. A Nd:YAG laser (532 nm, 50 mW) and 20 × objective were used for the measurements. Instrument calibration was achieved using a silicon reference. The data were directly collected from the powder samples in the Raman range of 80–620 cm^{-1} at room temperature. The Raman spectra of mixed Mo and W dichalcogenides Janus structures MX'X'' such as MoSSe, MoSeTe, MoSTe, WSSe, or WSeTe, have been reported in the literature.[40–44] The lack of correspondence between the experimental result and the data reported in the

literature suggests that such phases are not present in a significant proportion in the case of $MoW(SSeTe)_2$.

TEM samples were prepared by grinding bulk crystals with a size of ~ 2x2x1 mm^3 into small flakes in isopropanol in a ceramic mortar. The resulting dispersion was drop-casted onto Cu TEM grids coated with amorphous carbon films. Flakes with a size of ~ 300x300 nm^2 were identified in TEM and used for TEM analysis. TEM measurements were performed using a JEOL monochromated ARM200F microscope. The microscope is equipped with a double-Wien monochromator for the electron source, a CEOS ASCOR probe C_s corrector, a CEOS CETCOR image C_s corrector, a JEOL double silicon drift detector (SDD) for energy dispersive spectroscopy (EDX), a Continuum Gatan image filter (GIF) for electron energy loss spectroscopy (EELS), and high angle annular dark field (HAADF) scanning TEM (STEM) imaging detectors. The microscope was operated at 200 kV for atomic-resolution STEM HAADF imaging and STEM-EDX measurements. Atomic resolution STEM images were acquired with a pixel time of 4 µs and a pixel size of 512x512. The beam convergence half-angle and the HAADF inner collection-half-angle were 26.8 mrad and 54 mrad, respectively. 50 sequential STEM images were aligned and summed up to improve signal/noise ratio of the final images and were also used to monitor electron beam damage of the sample. STEM-EDX mapping was performed with a pixel size of 128x128 and effective dwell time of ~ 20 ms.

The surface composition of the samples was further studied with X-ray photoelectron spectroscopy (XPS) using SPECS spectrometer equipped with a monochromatic Al Kα X-ray source (1486.7 eV) and a hemispherical electron analyzer Phoibos 150. The survey spectra were recorded with E_p set to 100 eV, the high-resolution spectra of the core lines with E_p set to 20 eV. The base chamber pressure during the acquisitions was at 10^{-9} mbar or lower. Due to a heavy charge development on the sample surface, a low-energy electron flow generated by an electron flood gun has been used to compensate the charge buildup.

Inductively coupled plasma optical emission spectrometry (ICP-OES) measurement were performed with a Spectro ARCOS (SPECTRO Analytical Instruments). The spectrometer used the Paschen–Runge configuration with an optimized Rowland circle polychromator, measuring simultaneously in the broad spectral range of 130–770 nm using 32 linear CCD detectors. The detection limits are at parts per billion (by mass) levels for the elements determined. For the measurements, 5 mg of the the material was decomposed under microwave radiation in a mixture of H_2O_2, HNO_3, HF a $HClO_4$ and then determined. The evolved H_2 gas was monitored by gas chromatography (GC) equipped with a thermal conductivity detector (TCD) with Ar as a carrier gas (55977 MSD, Agilent Technologies).

Thermodinamic parameters calculation

Entropy of Mixing ΔS_{mix}

$$\Delta S_{mix} = -R\left[\left(\sum_{i=1}^{n} c_i ln\, c_i\right)_{cation} + \left(\sum_{j=1}^{n} c_j \ln c_j\right)_{anion}\right] \quad \text{(S1)}$$

ΔS_{mix} is the configurational entropy of the mixture; *R* is the the gas constant; *n* is the total number of component; c_{i} and c_{j} correspond to the molar proportions of components in the cation and anion positions, the coefficients represent the fractional contributions of metal (cation) and chalcogen (anion) sublattices, respectively. Conversion of ICP-OES wt.% to molar quantities obtained by atomic mass normalization.

Atomic size mismatch (δ)

$$\delta = \sqrt{\sum_{i=1}^{n} c_i(1 - r_i/\bar{r})^2} \quad \text{(S2)}$$

where *n* denotes the number of components, C_i and r_i denote the concentration and atomic radius of the *i*th component, respectively, and $\bar{r}$ is the average atomic size of the *n* components in the alloy. Atomic radius values can be obtained from online database, such as https://periodic-table.rsc.org/

Electronegativity difference (Δχ)

$$\Delta\chi = \sqrt{\sum_{i=1}^{n} c_i(\chi_i - \bar{\chi})^2} \quad \text{(S3)}$$

where *n* denotes the number of components, C_i and χ_i are the concentration and Pauling electronegativity of the *i*th component, respectively, and χ is the average electronegativity of the *n* components in the alloy. Electronegativity (Pauling scale) values can be obtained from online database, such as https://periodic-table.rsc.org/

Bond Length Variance (β)

$$\beta = \sqrt{\sum_{i=1}^{n} c_i \left(1 - \frac{r_{M-X(i)}}{\overline{r_{M-X}}}\right)^2} \tag{S4}$$

where n is the number of distinct bonds, c_i represents the molar fraction of component *i*, r_i denotes the M-X bond length for component *i*, and $\bar{r}_{M-X}$ signifies the weighted average bond length across all component. Visualization software such as Crystal Maker can be used to determine the average bond lengths of the structural units from databases like ICSD or https://next-gen.materialsproject.org/

XRD characterization

Obtained XRD was also used to analyze peak profiles within $10^o < 2\theta < 85^o$ the range for obtaining full-width at half-maximum (FWHM) value used for getting information about crystallite size. Each peak profile was fitted by the Lorentz curve which was shown to be better than the Gaussian curve which is more precise for XRD with a background without big noise. Peak center and FWHM were used for the calculation of particle size. The used method was the Scherrer equation:

$$B(2\theta) = \frac{K.\lambda}{L.\cos\theta} \tag{S5}$$

where B is peak wide at a value of 2θ, λ is X-ray wavelength, L is crystallite size and K is a constant generally around 1.0 for spherical particles. [45]

The diffraction pattern was decomposed using peak-fitting in order to obtain broad amorphous and sharp peaks from reflections of the crystalline peaks and to evaluate the crystallinity index (CI).

$$CI = A_c / A_t \tag{S6}$$

where A_c is the integrated area underneath the crystalline peaks and A_t represents the area of the total domain, both crystalline and amorphous regions, adjust baseline. [46]

Electrochemistry

i) Equations for rate determining steps in HER under acidic conditions (Tafel slopes)

Volmer step (120 mV/dec)

$$S + H^+ + e^- \rightarrow S - H_{ads} \tag{S7}$$

Heyrovsky step (40 mV/dec)

$$S - H_{ads} + H^+ + e^- \rightarrow S + H_2 \tag{S8}$$

Tafel step (30 mV/dec)

$$2(S - H_{ads}) \rightarrow 2S + H_2 \quad \text{(S9)}$$

ii) Tafel equation for Tafel slopes and j_0

Tafel equation: η = *b* log(j) + a; *b* corresponds to the Tafel slope.

Exchange current density (j_0) is calculated from when the overpotential (η) = 0 V.

$$j_0 = 10^{\frac{-a}{b}} \quad \text{(S10)}$$

iii) Mass Activity (MA)

$$MA = \frac{j\left(\frac{\text{mA}}{\text{cm}^2}\right)}{\text{mass of catalyst }\left(\frac{mg}{\text{cm}^2}\right)} \quad \text{(S11)}$$

iv) Double-layer capacitance (C_{dl}) measurements were carried out in purged medium in a region with no faradaic process, in 1.0 M KOH. Different scan rates (ν = 10,20, 50, 100, 150, 200, 250, 300 mV s^{-1}) were applied and the capacitance was extracted from the slope of the dependence of current density ($\Delta I_a - I_c$) with the scan rate at a fixed potential of +0.723 V vs. RHE. Estimation of C_{dl} from CVs data shown in Figure S5.

$$C_{dl} = \frac{|j_a - j_c|}{2\nu} \quad \text{(S12)}$$

v) Circuit for fitting of EIS data and estimation of R_{ct} , [R(RQ)(RQ)]. The electrochemical impedance spectroscopy (EIS) for measurement of R_{ct} response was carried out in the same media as HER measurements at −0.095 V vs. RHE with a frequency ranging from 1 MHz to 0.1 Hz and an amplitude of 10 mV.

vi) Turnover frequency (TOF) calculations of the TMD_{mix}, exf-TMD_{big} and exf-TMD_{small} were done following the proposed formulas [47,48]

$$\text{TOF }(\text{H}_2/\text{s}) = \frac{\text{n H}_2\text{ tunrovers/cm}^2}{\text{n active site / cm}^2} \quad \text{(S13)}$$

The number of total hydrogen turnover was calculated from *I* in the LSVs

$$n\,\text{H}_2\frac{\text{tunrovers}}{\text{cm}^2} = \left(\frac{\text{mA}}{\text{cm}^2}\right)\cdot\left(\frac{1\text{ mol e}^-}{96485.3\text{ C}}\right)\cdot\left(\frac{1\text{ mol}}{2\text{ mol e}^-}\right)\cdot\left(\frac{6.022\text{x}10^{23}\text{ molecules H}_2}{\text{mol H}_2}\right)$$
$$= 3.12\text{x}10^{15}\frac{\text{H}_2/\text{s}}{\text{cm}^2}\text{ per }\frac{\text{mA}}{\text{cm}^2} \quad \text{(S14)}$$

The of actives sites in the each material were calculated from the content of MoW as determined by EDX quantification, considering each metallic MoW atoms as an active site

$$\text{MoW sites/cm}^2 = \left(\frac{\text{MoW loading}\left(\frac{\text{g}}{\text{cm}^2}\right).\ \text{MoW}\ wt.\%}{\text{MoW}\ M_w\ \left(\frac{\text{g}}{\text{mol}}\right)}\right).\left(\frac{\text{molecules MoW}}{\text{mol MoW}}\right) \quad \text{(S15)}$$

For the Faradaic efficiency (FE) experiment, an air tight H-type cell was used, with a nafion membrane (NR-211, Ion Power GmbH) between cathode and anode. The volume of the electrolyte (0.5 M H_2SO_4) in the anode and the cathode was 50 mL for each, Throughout, a magnetic stirring at 400 rpm was applied for removal of gas bubbles, and performed at room temperature (25 °C), The WE and RE were placed at the cathode and the CE at the anode. The gas produced at the cathode was collect, either at an inverted graduated proved for the gas displacement experiment, or at a GC syringe for GC-TCD measurement (55977 MSD, Agilent Technologies). The FE for a water-splitting reaction, specifically for H_2 evolution, is a measure of how effectively the applied charge (electric current) is used to produce hydrogen gas. It is calculated using the following formula:

$$FE = \frac{n\,H_2\ experimental\ (\mu mol)}{n\,H_2\ \ theory\ (\mu mol)} \quad \text{(S16)}$$

The theoretical quantity of H_2 gas can be calculated from the total charge, Q (C), passed during the electrolysis. This relates to the number of moles of H_2 gas evolved from the cathode, through Faraday's laws:

$$n\,H_2\ \ theory = \frac{I.t}{2e^-.F} \quad \text{(S17)}$$

Where 2 is the number of e^- required to reduce 2 H^+ ions to 1 molecule of H_2 (g), and the Faraday's constant is 96485 C/mol. For this experiment, an H-type cell was used, with collection of the gas produced at the cathode, using an electrode with 2 cm^2, at 10 mA/cm^2, for 60 min. The number of moles of H_2 theoretically expected is 0.373 mmol. Using the water displacement method, the volume of H_2 collected from the cathode can be converted considering that the molar volume of any gas at Standard Temperature and Pressure (STP) is 22.4 L.

$$n\,H_2\ \ exp = \frac{V\,H_2\ exp}{22.4\ L/mol} \quad \text{(S18)}$$

The average collected was 7.9 ± 0.4 mL, for the same above mentioned conditions. From this value, the experimental number of moles obtained was 0.353 ± 0.017 mmol H_2, or 0.177 ± 0.009 mmol H_2 h^{-1} cm^{-2}, corresponding to a Faradaic efficiency (FE) of 94.7 ± 4.6 %.

References

(1) Allen, L. J.; D'Alfonso, A. J.; Findlay, S. D. Modelling the Inelastic Scattering of Fast Electrons. *Ultramicroscopy* **2015**, *151*, 11–22. https://doi.org/10.1016/j.ultramic.2014.10.011.

(2) Qiu, H. J.; Fang, G.; Wen, Y.; Liu, P.; Xie, G.; Liu, X.; Sun, S. Nanoporous High-Entropy Alloys for Highly Stable and Efficient Catalysts. *J. Mater. Chem. A* **2019**, *7* (11), 6499–6506. https://doi.org/10.1039/C9TA00505F.

(3) He, H.-Y. Metallic WSe2: Sn Nanosheets Assembled on Graphene by a Modified Hydrothermal Process for Hydrogen Evolution Reaction. *Colloids Surfaces A Physicochem. Eng. Asp.* **2020**, *589*, 124149. https://doi.org/10.1016/j.colsurfa.2019.124149.

(4) Luo, Y.; Li, X.; Cai, X.; Zou, X.; Kang, F.; Cheng, H.-M.; Liu, B. Two-Dimensional MoS2 Confined Co(OH)2 Electrocatalysts for Hydrogen Evolution in Alkaline Electrolytes. *ACS Nano* **2018**, *12* (5), 4565–4573. https://doi.org/10.1021/acsnano.8b00942.

(5) He, H.-Y.; He, Z.; Shen, Q. TiO 2 : Si Nanotube/1T-MoSe 2 Nanosheet Hybrids with Highly Efficient Hydrogen Evolution Catalytic Activity. *J. Colloid Interface Sci.* **2018**, *522*, 136–143. https://doi.org/10.1016/j.jcis.2018.03.068.

(6) Yu, Q.; Luo, Y.; Qiu, S.; Li, Q.; Cai, Z.; Zhang, Z.; Liu, J.; Sun, C.; Liu, B. Tuning the Hydrogen Evolution Performance of Metallic 2d Tantalum Disulfide by Interfacial Engineering. *ACS Nano* **2019**, *13* (10), 11874–11881. https://doi.org/10.1021/ACSNANO.9B05933.

(7) Zhang, G.; Ming, K.; Kang, J.; Huang, Q.; Zhang, Z.; Zheng, X.; Bi, X. High Entropy Alloy as a Highly Active and Stable Electrocatalyst for Hydrogen Evolution Reaction. *Electrochim. Acta* **2018**, *279*, 19–23. https://doi.org/10.1016/j.electacta.2018.05.035.

(8) He, Q.; Wang, L.; Yin, K.; Luo, S. Vertically Aligned Ultrathin 1T-WS2 Nanosheets Enhanced the Electrocatalytic Hydrogen Evolution. *Nanoscale Res. Lett.* **2018**, *13* (1), 167. https://doi.org/10.1186/s11671-018-2570-x.

(9) Wang, R.; Huang, J.; Zhang, X.; Han, J.; Zhang, Z.; Gao, T.; Xu, L.; Liu, S.; Xu, P.; Song, B. Two-Dimensional High-Entropy Metal Phosphorus Trichalcogenides for Enhanced Hydrogen Evolution Reaction. *ACS Nano* **2022**, *16* (3), 3593–3603. https://doi.org/10.1021/acsnano.2c01064.

(10) Kunitski, M.; Eicke, N.; Huber, P.; Köhler, J.; Zeller, S.; Voigtsberger, J.; Schlott, N.; Henrichs, K.; Sann, H.; Trinter, F.; Schmidt, L. P. H.; Kalinin, A.; Schöffler, M. S.; Jahnke, T.; Lein, M.; Dörner, R. Double-Slit Photoelectron Interference in Strong-Field Ionization of the Neon Dimer. *Nat. Commun.* **2019**, *10* (1), 1. https://doi.org/10.1038/s41467-018-07882-8.

(11) Zhou, W. Y.; Li, S. S.; Xiao, X. Y.; Chen, S. H.; Liu, J. H.; Huang, X. J. Defect- and Phase-Engineering of Mn-Mediated MoS2 Nanosheets for Ultrahigh Electrochemical Sensing of Heavy Metal Ions: Chemical Interaction-Driven in Situ Catalytic Redox Reactions. *Chem. Commun.* **2018**, *54* (67), 9329–9332. https://doi.org/10.1039/C8CC04575E.

(12) Karfa, P.; Majhi, K. C.; Madhuri, R. Group IV Transition Metal Based Phospho-Chalcogenides@MoTe2

for Electrochemical Hydrogen Evolution Reaction over Wide Range of PH. *Int. J. Hydrogen Energy* **2019**, *44* (45), 24628–24641. https://doi.org/10.1016/J.IJHYDENE.2019.07.192.

(13) Liu, Z.; Zhao, L.; Liu, Y.; Gao, Z.; Yuan, S.; Li, X.; Li, N.; Miao, S. Vertical Nanosheet Array of 1T Phase MoS2 for Efficient and Stable Hydrogen Evolution. *Appl. Catal. B Environ.* **2019**, *246*, 296–302. https://doi.org/10.1016/J.APCATB.2019.01.062.

(14) Yin, Y.; Zhang, Y.; Gao, T.; Yao, T.; Zhang, X.; Han, J.; Wang, X.; Zhang, Z.; Xu, P.; Zhang, P.; Cao, X.; Song, B.; Jin, S.; Yin, Y.; Zhang, Y.; Zhang, X.; Han, J.; Yao, T.; Wang, X.; Song, B.; Zhang, Z.; Xu, P.; Zhang, P.; Cao, X.; Jin, S. Synergistic Phase and Disorder Engineering in 1T-MoSe2 Nanosheets for Enhanced Hydrogen-Evolution Reaction. *Adv. Mater.* **2017**, *29* (28), 1700311. https://doi.org/10.1002/ADMA.201700311.

(15) Huang, C.; Wang, X.; Wang, D.; Zhao, W.; Bu, K.; Xu, J.; Huang, X.; Bi, Q.; Huang, J.; Huang, F. Atomic Pillar Effect in Pd XNbS2 to Boost Basal Plane Activity for Stable Hydrogen Evolution. *Chem. Mater.* **2019**, *31* (13), 4726–4731. https://doi.org/10.1021/ACS.CHEMMATER.9B00821.

(16) Zhang, Q.; Chen, W.; Chen, G.; Huang, J.; Song, C.; Chu, S.; Zhang, R.; Wang, G.; Li, C.; Ostrikov, K. K. Bi-Metallic Nitroxide Nanodot-Decorated Tri-Metallic Sulphide Nanosheets by on-Electrode Plasma-Hydrothermal Sprouting for Overall Water Splitting. *Appl. Catal. B Environ.* **2020**, *261*, 118254. https://doi.org/10.1016/j.apcatb.2019.118254.

(17) Voiry, D.; Yamaguchi, H.; Li, J.; Silva, R.; Alves, D. C. B.; Fujita, T.; Chen, M.; Asefa, T.; Shenoy, V. B.; Eda, G.; Chhowalla, M. Enhanced Catalytic Activity in Strained Chemically Exfoliated WS2 Nanosheets for Hydrogen Evolution. *Nat. Mater. 2013 129* **2013**, *12* (9), 850–855. https://doi.org/10.1038/nmat3700.

(18) Huang, N.; Peng, R.; Ding, Y.; Yan, S.; Li, G.; Sun, P.; Sun, X.; Liu, X.; Yu, H. Facile Chemical-Vapour-Deposition Synthesis of Vertically Aligned Co-Doped MoS2 Nanosheets as an Efficient Catalyst for Triiodide Reduction and Hydrogen Evolution Reaction. *J. Catal.* **2019**, *373*, 250–259. https://doi.org/10.1016/J.JCAT.2019.04.007.

(19) Lukowski, M. A.; Daniel, A. S.; Meng, F.; Forticaux, A.; Li, L.; Jin, S. Enhanced Hydrogen Evolution Catalysis from Chemically Exfoliated Metallic MoS 2 Nanosheets. *J. Am. Chem. Soc.* **2013**, *135* (28), 10274–10277. https://doi.org/10.1021/ja404523s.

(20) Yin, Y.; Han, J.; Zhang, Y.; Zhang, X.; Xu, P.; Yuan, Q.; Samad, L.; Wang, X.; Wang, Y.; Zhang, Z.; Zhang, P.; Cao, X.; Song, B.; Jin, S. Contributions of Phase, Sulfur Vacancies, and Edges to the Hydrogen Evolution Reaction Catalytic Activity of Porous Molybdenum Disulfide Nanosheets. *J. Am. Chem. Soc.* **2016**, *138* (25), 7965–7972. https://doi.org/10.1021/jacs.6b03714.

(21) Han, G. Q.; Liu, Y. R.; Hu, W. H.; Dong, B.; Li, X.; Chai, Y. M.; Liu, Y. Q.; Liu, C. G. WS2 Nanosheets Based on Liquid Exfoliation as Effective Electrocatalysts for Hydrogen Evolution Reaction. *Mater. Chem. Phys.* **2015**, *167*, 271–277. https://doi.org/10.1016/J.MATCHEMPHYS.2015.10.043.

(22) Huan, Y.; Shi, J.; Zou, X.; Gong, Y.; Zhang, Z.; Li, M.; Zhao, L.; Xu, R.; Jiang, S.; Zhou, X.; Hong, M.; Xie, C.; Li, H.; Lang, X.; Zhang, Q.; Gu, L.; Yan, X.; Zhang, Y. Vertical 1T-TaS 2 Synthesis on Nanoporous Gold for High-Performance Electrocatalytic Applications. *Adv. Mater.* **2018**, *30* (15), 1705916. https://doi.org/10.1002/adma.201705916.

(23) Wang, J.; Tang, J.; Guo, T.; Zhang, S.; Xia, W.; Tan, H.; Bando, Y.; Wang, X.; Yamauchi, Y. C3N4-Digested 3D Construction of Hierarchical Metallic Phase MoS2 Nanostructures. *J. Mater. Chem. A*

2019, *7* (31), 18388–18396. https://doi.org/10.1039/C9TA06115K.

(24) Huang, Y.; Ma, Z.; Hu, Y.; Chai, D.; Qiu, Y.; Gao, G.; Hu, P. An Efficient WSe2/Co0.85Se/Graphene Hybrid Catalyst for Electrochemical Hydrogen Evolution Reaction. *RSC Adv.* **2016**, *6* (57), 51725–51731. https://doi.org/10.1039/C6RA08618G.

(25) Wang, K.; Ye, Z.; Liu, C.; Xi, D.; Zhou, C.; Shi, Z.; Xia, H.; Liu, G.; Qiao, G. Morphology-Controllable Synthesis of Cobalt Telluride Branched Nanostructures on Carbon Fiber Paper as Electrocatalysts for Hydrogen Evolution Reaction. *ACS Appl. Mater. Interfaces* **2016**, *8* (5), 2910–2916. https://doi.org/10.1021/ACSAMI.5B10835.

(26) Lu, D.; Ren, X.; Ren, L.; Xue, W.; Liu, S.; Liu, Y.; Chen, Q.; Qi, X.; Zhong, J. Direct Vapor Deposition Growth of 1T′ MoTe2on Carbon Cloth for Electrocatalytic Hydrogen Evolution. *ACS Appl. Energy Mater.* **2020**, *3* (4), 3212–3219. https://doi.org/10.1021/ACSAEM.9B01589.

(27) Chen, T. T.; Wang, R.; Li, L. K.; Li, Z. J.; Zang, S. Q. MOF-Derived Co9S8/MoS2 Embedded in Tri-Doped Carbon Hybrids for Efficient Electrocatalytic Hydrogen Evolution. *J. Energy Chem.* **2020**, *44*, 90–96. https://doi.org/10.1016/J.JECHEM.2019.09.018.

(28) Yang, L.; Zhou, W.; Hou, D.; Zhou, K.; Li, G.; Tang, Z.; Li, L.; Chen, S. Porous Metallic MoO2-Supported MoS2 Nanosheets for Enhanced Electrocatalytic Activity in the Hydrogen Evolution Reaction. *Nanoscale* **2015**, *7* (12), 5203–5208. https://doi.org/10.1039/C4NR06754A.

(29) Lu, T. H.; Chen, C. J.; Basu, M.; Ma, C. G.; Liu, R. S. The CoTe2 Nanostructure: An Efficient and Robust Catalyst for Hydrogen Evolution. *Chem. Commun.* **2015**, *51* (95), 17012–17015. https://doi.org/10.1039/C5CC06806A.

(30) Xiao, W.; Li, Y.; Elgendy, A.; Duran, E. C.; Buckingham, M. A.; Spencer, B. F.; Han, B.; Alam, F.; Zhong, X.; Cartmell, S. H.; Cernik, R. J.; Eggeman, A. S.; Dryfe, R. A. W.; Lewis, D. J. Synthesis of High Entropy and Entropy-Stabilized Metal Sulfides and Their Evaluation as Hydrogen Evolution Electrocatalysts. *Chem. Mater.* **2023**, *35* (19), 7904–7914. https://doi.org/10.1021/acs.chemmater.3c00363.

(31) Li, H.; Qian, X.; Xu, C.; Huang, S.; Zhu, C.; Jiang, X.; Shao, L.; Hou, L. Hierarchical Porous Co 9 S 8 /Nitrogen-Doped Carbon@MoS 2 Polyhedrons as PH Universal Electrocatalysts for Highly Efficient Hydrogen Evolution Reaction. *ACS Appl. Mater. Interfaces* **2017**, *9* (34), 28394–28405. https://doi.org/10.1021/acsami.7b06384.

(32) Wang, H.; Kong, D.; Johanes, P.; Cha, J. J.; Zheng, G.; Yan, K.; Liu, N.; Cui, Y. MoSe2 and WSe2 Nanofilms with Vertically Aligned Molecular Layers on Curved and Rough Surfaces. *Nano Lett.* **2013**, *13* (7), 3426–3433. https://doi.org/10.1021/nl401944f.

(33) Li, J.; Zhou, Q.; Yuan, C.; Cheng, P.; Hu, X.; Huang, W.; Gao, X.; Wang, X.; Jin, M.; Nötzel, R.; Zhou, G.; Zhang, Z.; Liu, J. Direct Growth of Vertically Aligned ReSe2 Nanosheets on Conductive Electrode for Electro-Catalytic Hydrogen Production. *J. Colloid Interface Sci.* **2019**, *553*, 699–704. https://doi.org/10.1016/J.JCIS.2019.06.073.

(34) Jiang, S.; Zhang, Z.; Zhang, N.; Huan, Y.; Gong, Y.; Sun, M.; Shi, J.; Xie, C.; Yang, P.; Fang, Q.; Li, H.; Tong, L.; Xie, D.; Gu, L.; Liu, P.; Zhang, Y. Application of Chemical Vapor–Deposited Monolayer ReSe2 in the Electrocatalytic Hydrogen Evolution Reaction. *Nano Res.* **2018**, *11* (4), 1787–1797. https://doi.org/10.1007/S12274-017-1796-8.

(35) Regner, J.; Mourdikoudis, S.; Gusmão, R.; Sofer, Z. MoS2 Nanoensembles Prepared by a Simple

Solvothermal Route for Hydrogen Evolution Reaction. *FlatChem* **2023**, *42*, 100566. https://doi.org/10.1016/j.flatc.2023.100566.

(36) Du, C.; Liang, D.; Shang, M.; Zhang, J.; Mao, J.; Liu, P.; Song, W. In Situ Engineering MoS2 NDs/VS2 Lamellar Heterostructure for Enhanced Electrocatalytic Hydrogen Evolution. *ACS Sustain. Chem. Eng.* **2018**, *6* (11), 15471–15479. https://doi.org/10.1021/acssuschemeng.8b03929.

(37) Sokolikova, M. S.; Sherrell, P. C.; Palczynski, P.; Bemmer, V. L.; Mattevi, C. Direct Solution-Phase Synthesis of 1T' WSe2 Nanosheets. *Nat. Commun.* **2019**, *10* (1), 712. https://doi.org/10.1038/s41467-019-08594-3.

(38) Wang, Z.; Zhang, L. Nickel Ditelluride Nanosheet Arrays: A Highly Efficient Electrocatalyst for the Oxygen Evolution Reaction. *ChemElectroChem* **2018**, *5* (8), 1153–1158. https://doi.org/10.1002/CELC.201701357.

(39) Seok, J.; Lee, J.-H.; Bae, D.; Ji, B.; Son, Y.-W.; Lee, Y. H.; Yang, H.; Cho, S. Hybrid Catalyst with Monoclinic MoTe2 and Platinum for Efficient Hydrogen Evolution. *APL Mater.* **2019**, *7* (7), 071118. https://doi.org/10.1063/1.5094957.

(40) Petrić, M. M.; Kremser, M.; Barbone, M.; Qin, Y.; Sayyad, Y.; Shen, Y.; Tongay, S.; Finley, J. J.; Botello-Méndez, A. R.; Müller, K. Raman Spectrum of Janus Transition Metal Dichalcogenide Monolayers WSSe and MoSSe. *Phys. Rev. B* **2021**, *103* (3), 035414. https://doi.org/10.1103/PhysRevB.103.035414.

(41) Li, P.; Cui, J.; Zhou, J.; Guo, D.; Zhao, Z.; Yi, J.; Fan, J.; Ji, Z.; Jing, X.; Qu, F.; Yang, C.; Lu, L.; Lin, J.; Liu, Z.; Liu, G.; Yi, J.; Ji, Z.; Jing, X.; Liu, G.; Yang, C.; Lu, L.; Lin, J.; Li, P.; Cui, J.; Guo, D.; Zhao, Z.; Fan, J.; Qu, F.; Zhou, J.; Liu, Z. Phase Transition and Superconductivity Enhancement in Se-Substituted MoTe2 Thin Films. *Adv. Mater.* **2019**, *31* (48), 1904641. https://doi.org/10.1002/ADMA.201904641.

(42) Yagmurcukardes, M.; Sevik, C.; Peeters, F. M. Electronic, Vibrational, Elastic, and Piezoelectric Properties of Monolayer Janus MoSTe Phases: A First-Principles Study. *Phys. Rev. B* **2019**, *100* (4), 045415. https://doi.org/10.1103/PhysRevB.100.045415.

(43) Mehdipour, H.; Kratzer, P. First-Principles Calculations of MoSeTe/WSeTe Bilayers: Stability, Phonons, Electronic Band Offsets, and Rashba Splitting. *Phys. Rev. B* **2024**, *109* (8), 085425. https://doi.org/10.1103/PhysRevB.109.085425.

(44) Serra, M.; Lajaunie, L.; Sreedhara, M. B.; Miroshnikov, Y.; Pinkas, I.; Calvino, J. J.; Enyashin, A. N.; Tenne, R. Quaternary LnxLa(1-x)S-TaS2 Nanotubes (Ln=Pr, Sm, Ho, and Yb) as a Vehicle for Improving the Yield of Misfit Nanotubes. *Appl. Mater. Today* **2020**, *19*, 100581. https://doi.org/10.1016/j.apmt.2020.100581.

(45) Patterson, A. L. The Scherrer Formula for X-Ray Particle Size Determination. *Phys. Rev.* **1939**, *56* (10), 978. https://doi.org/10.1103/PhysRev.56.978.

(46) Navarro-Pardo, F.; Martínez-Barrera, G.; Martínez-Hernández, A. L.; Castaño, V. M.; Rivera-Armenta, J. L.; Medellín-Rodríguez, F.; Velasco-Santos, C. Effects on the Thermo-Mechanical and Crystallinity Properties of Nylon 6,6 Electrospun Fibres Reinforced with One Dimensional (1D) and Two Dimensional (2D) Carbon. *Materials (Basel).* **2013**, *6* (8), 3494–3513. https://doi.org/10.3390/ma6083494.

(47) Kuang, P.; Wang, Y.; Zhu, B.; Xia, F.; Tung, C. W.; Wu, J.; Chen, H. M.; Yu, J. Pt Single Atoms

Supported on N-Doped Mesoporous Hollow Carbon Spheres with Enhanced Electrocatalytic H2-Evolution Activity. *Adv. Mater.* **2021**, *33* (18), 2008599. https://doi.org/10.1002/adma.202008599.

(48) Fei, H.; Dong, J.; Arellano-Jiménez, M. J.; Ye, G.; Dong Kim, N.; Samuel, E. L. G.; Peng, Z.; Zhu, Z.; Qin, F.; Bao, J.; Yacaman, M. J.; Ajayan, P. M.; Chen, D.; Tour, J. M. Atomic Cobalt on Nitrogen-Doped Graphene for Hydrogen Generation. *Nat. Commun.* **2015**, *6* (1), 1–8. https://doi.org/10.1038/ncomms9668.
